# Procrustes is a machine-learning approach that removes cross-platform batch effects from clinical RNA sequencing data

**DOI:** 10.1038/s42003-024-06020-z

**Published:** 2024-03-30

**Authors:** Nikita Kotlov, Kirill Shaposhnikov, Cagdas Tazearslan, Madison Chasse, Artur Baisangurov, Svetlana Podsvirova, Dawn Fernandez, Mary Abdou, Leznath Kaneunyenye, Kelley Morgan, Ilya Cheremushkin, Pavel Zemskiy, Maxim Chelushkin, Maria Sorokina, Ekaterina Belova, Svetlana Khorkova, Yaroslav Lozinsky, Katerina Nuzhdina, Elena Vasileva, Dmitry Kravchenko, Kushal Suryamohan, Krystle Nomie, John Curran, Nathan Fowler, Alexander Bagaev

**Affiliations:** BostonGene, Corp., Waltham, MA 02453 USA

**Keywords:** Machine learning, Molecular medicine, Bioinformatics

## Abstract

With the increased use of gene expression profiling for personalized oncology, optimized RNA sequencing (RNA-seq) protocols and algorithms are necessary to provide comparable expression measurements between exome capture (EC)-based and poly-A RNA-seq. Here, we developed and optimized an EC-based protocol for processing formalin-fixed, paraffin-embedded samples and a machine-learning algorithm, Procrustes, to overcome batch effects across RNA-seq data obtained using different sample preparation protocols like EC-based or poly-A RNA-seq protocols. Applying Procrustes to samples processed using EC and poly-A RNA-seq protocols showed the expression of 61% of genes (*N* = 20,062) to correlate across both protocols (concordance correlation coefficient > 0.8, versus 26% before transformation by Procrustes), including 84% of cancer-specific and cancer microenvironment-related genes (versus 36% before applying Procrustes; *N* = 1,438). Benchmarking analyses also showed Procrustes to outperform other batch correction methods. Finally, we showed that Procrustes can project RNA-seq data for a single sample to a larger cohort of RNA-seq data. Future application of Procrustes will enable direct gene expression analysis for single tumor samples to support gene expression-based treatment decisions.

## Abstract (web summary)

Procrustes is a machine-learning algorithm that can overcome batch effects across RNA-seq data obtained by different sample preparation methods, like exome capture-based or poly-A RNA-seq protocols.

## Introduction

RNA sequencing (RNA-seq) for gene expression profiling (GExP) has been reported as a powerful tool that is widely used in oncology research^1^. It is being increasingly implemented in clinical settings for a wide variety of applications, including for biomarker discovery, predicting prognosis, and guiding the use of adjuvant therapy^1^. Until recently, implementing high-throughput RNA-seq in the clinic, however, has been a challenge due to the need for fresh-frozen tumor samples to obtain optimal results. In the clinical setting, specimens are predominantly preserved as formalin-fixed, paraffin-embedded (FFPE) tissues for long-term storage. This preservation process leads to rapid degradation in RNA quality^2^. Poly-A RNA-seq, on the other hand, is stable, reproducible, and one of the most widely used methods for GExP. Unfortunately, it requires fresh or freshly frozen (FF) tissues and poorly captures partially degraded mRNAs, thus rendering it unsuitable for use in the clinical setting.

Exome capture (EC)-based RNA-seq protocols provide better-quality data for RNA-seq of FFPE tissues and are now routinely used in the clinic^2^. EC-based RNA-seq differs from poly-A RNA-seq in that it is vendor-dependent and is highly customizable for targeting different gene sets, resulting in cohorts sequenced using different protocols and targeted gene panels. This situation presents several challenges, including batch effects and the compatibility of datasets generated by different sequencing chemistries and technologies or platforms, complicating statistical analyses. While sample quality has been reported as a contributor to batch effects, it alone cannot explain many other batch effects that occur when comparing different samples within a dataset or across different datasets^3^. The comparison and integration of data from different sequencing protocols such as poly-A and EC further exacerbates this complication. For example, the Cancer Genome Atlas (TCGA) has established a database of well-annotated poly-A RNA-sequenced samples from FF tissues for more than 30 cancer types, creating a valuable resource of sequencing data that can potentially be utilized in comparative GExP studies as previously shown^4^. While we previously successfully integrated poly-A RNA-seq from different studies without any correction, the utilization of different sequencing protocols necessitates correction^4^. However, comparing or integrating such data with FFPE-derived EC-based data can be challenging because of technical batch effects that arise due to variation and differences across different protocols. While differences in the overall distribution of gene expression profiles within individual samples can be corrected by normalization, batch effects arising from differences in sequencing protocols cannot be eliminated using conventional approaches^5,6^. Although several studies have demonstrated concordance between FF- and FFPE-derived poly-A RNA-seq data^6^, there is still a need to develop sequencing protocols and data processing algorithms that allow direct comparison of gene expression across different sequencing protocols (EC-based and poly-A RNA-seq) and overcome any underlying batch effects.

Several batch correction methods are available for RNA-seq data analysis, including negative binomial regression models (ComBat-Seq), surrogate variable analysis (SVA), dimensionality reduction techniques (such as self-supervised contrastive learning (CLEAR)^7^), and normalization-based methods (such as Z-scoring and BMC). However, none of these can be applied to an individual sample for comparing expression values to a cohort^6,8–10^. Thus, there is an unmet need to develop an algorithm for batch effect correction of multi-platform data and for projecting the expression values of a single sample to a larger cohort in order to improve gene expression-based personalized clinical decision-making.

To address this unmet need, we developed an optimized EC-based RNA sequencing protocol and Procrustes, a batch correction machine learning (ML) algorithm that enables the projection of RNA-seq data from individual samples onto a cohort of samples sequenced using different methodologies. Specifically, we demonstrated the capability of our algorithm to minimize batch effects when comparing RNA-seq data obtained using EC-based and poly-A-based protocols. This workflow enables the unification of datasets produced using different sequencing protocols and fills a major gap in the field of computational biology.

## Results

### An improved EC-based protocol for FFPE RNA-seq

EC-based RNA-seq is increasingly being utilized for GExP in clinical settings to overcome the challenges in obtaining RNA of sufficient integrity from clinical specimens. To more closely recapitulate poly-A-based RNA-seq protocols, we sought to optimize an EC-based RNA-seq protocol by modifying the Agilent XT HS2 V7 probe set. Briefly, we included probes to the 5’ and 3’ UTR regions to better mimic poly-A RNA-seq gene expression distribution profiles with more uniform 5’ to 3’ gene body coverage (see Methods). Using 28 biopsy specimens processed as FF and FFPE technical replicates, we generated RNA-seq data using our modified EC (Agilent XT HS2 V7 UTR), Agilent XT HS2 V7, and poly-A RNA-seq protocols (Supplementary Data 1, Fig. 1a; see Methods). Libraries from both the modified EC and poly-A RNA-seq protocols showed high alignment rates with a low percentage of unmapped reads (Supplementary Fig. 1a; Supplementary Data 2).

**Fig. 1:**
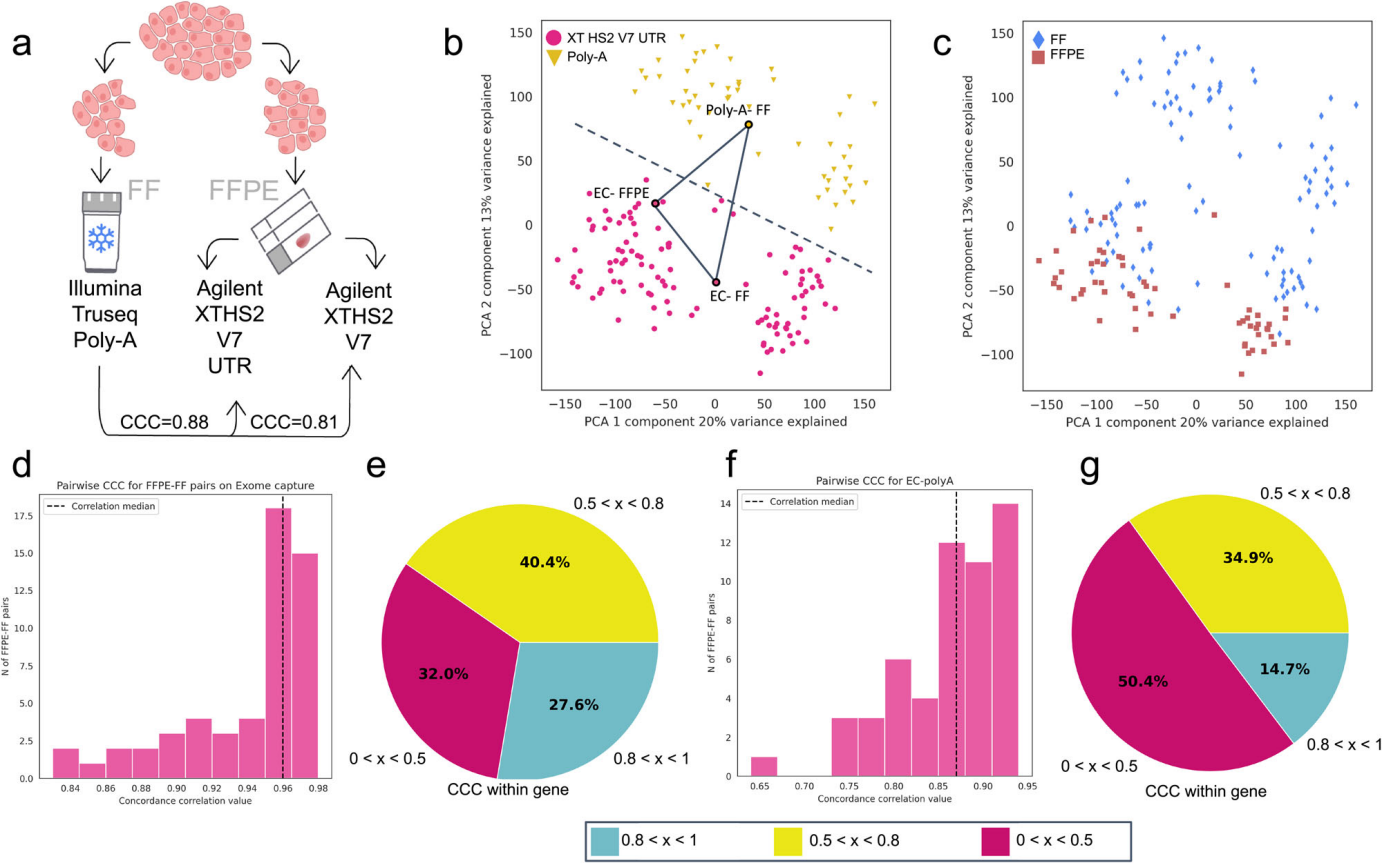
Optimization of exome capture-based (EC) RNA-seq protocol. **a** Schematic of EC-protocol optimization. Median CCC for V7 versus V7 UTR. **b** Principal Component Analysis (PCA) showing batch effect between the modified EC (V7 UTR) and poly-A RNA-seq protocols. **c** PCA showing the absence of batch effect between samples stored as either FF or FFPE and sequenced with the modified EC protocol. **d** Distribution of within-sample CCC between FF and FFPE samples processed using the modified EC protocol. **e** Pie chart showing percentage of genes with low (<0.5), medium (0.5-0.8), and high (>0.8) within-gene CCC between samples stored as FF or FFPE and processed using the modified EC protocol. **f** Distribution of within-sample CCC for pairwise comparison between FF (poly-A) and FFPE (EC) samples. **g** Pie chart showing percentage of genes with low (<0.5), medium (0.5–0.8), and high (>0.8) within-gene CCC between modified EC and poly-A protocols. CCC concordance correlation coefficient, EC exome capture.

Next, we assessed the concordance of gene expression between the modified EC-based protocol and poly-A RNA-seq. The concordance correlation coefficient (CCC) was chosen as the main parameter measured because it can assess if the corresponding values in two vectors are equal to each other^11^. Our analysis showed that the modified EC-based protocol performed better in recapitulating poly-A RNA-seq data than the unmodified Agilent XT HS2 V7 probe set, improving the median CCC calculated for all 20,062 genes analyzed from 0.81 to 0.88. (Fig. 1a, Supplementary Fig. 1a–d, Supplementary Data 2). However, despite the improved performance, we still observed pronounced batch effects when comparing data from FF sample-derived poly-A libraries to modified EC libraries from both FF and FFPE samples, indicating that batch effects likely arose not from differences in sample storage methods, but from differences in the library preparation protocols (Fig. 1b, c). We repeated our analysis using human cell lines and sorted cell populations to confirm that the observed batch effects were a result of differences in library preparation methods (Supplementary Data 3). Batch effects were assessed by considering each sample type separately in order to confirm that these results were not confounded by sample source. Consistent with the tissue-based results, a batch effect that separated the data by library preparation protocol used was also present in samples of human cell lines and sorted cell populations (Fig. 1b, Supplementary Fig. 1e–i; see Methods). To further confirm that batch effects were a result of differences in library preparation protocols, we used the modified EC-based protocol to process each sample as FF and FFPE replicates (Supplementary Data 4). We observed a high median CCC of 0.950 across the transcriptome (~20,000 genes) and minimal batch effect between both storage methods in pairwise comparison (Fig. 1d, Supplementary Data 5). Here, at the individual gene level, 27.6% of genes had CCC values higher than 0.8 (Fig. 1e) compared to 14.6% for the poly-A versus EC comparison (Fig. 1f, g), suggesting that the persistent bath effects at the individual gene level are due to differences in library preparation protocols and not differences in sample storage protocols, thus necessitating the need for an approach to overcome these batch effects.

### Limitations of existing batch effect correction methods

To evaluate the ability of currently available tools to overcome protocol-specific batch effects, we examined the performance of several algorithms including DASC^12^, Z-scores and BMC normalization methods, Random Forest Regression, Ridge and Lasso linear regression, ComBat-Seq^6^, and a Mutual Nearest Neighbors (MNN)-based model^13^, as described in Table 1. Here, we chose to correct samples from the publicly available MET500 cohort (phs000673.v2.p1)^6,14–16^. This comprehensive cohort encompasses samples of diverse cancer diagnoses sequenced as replicates using both poly-A RNA-seq and Agilent Sureselect V4 EC protocols (Supplementary Fig. 1j), thus allowing us to demonstrate the utility and versatility of Procrustes in our benchmarking approach. Among the 360 paired samples considered for this analysis, 296 paired samples passed quality control measures (Supplementary Fig. 2a, see Methods). Next, the chosen MET500 cohort samples were divided into training (*N* = 181) and holdout (*N* = 115) datasets (Supplementary Fig. 2b, c; Supplementary Data 6).

**Table 1:** List of algorithms chosen as benchmarking targets for Procrustes

Batch correction method	Description	Median CCC	References
ComBat-Seq	One of the most widely used batch correction algorithms that utilizes a negative binomial regression	0.72	^6^
Mutual nearest neighbors (MNN)	Commonly used for batch correction for single-cell (sc)RNA-seq	0.62 for MNN and 0.76 for DASC	^12,13^
DASC			
Random forest regression	Ensemble decision trees method, used to assess non-linearities in our benchmarking approach	0.75	^60^
Z-scores	Normalization batch correction techniques	0.72 for Z-scores and 0.83 for BMC	^61,62^
BMC			
Ridge regression	Evaluated separately in our benchmarking approach to compare the performance of either l1 or l2 penalization alone (Procrustes) with the performance of combined l1/l2 penalization utilized within ElasticNet (Ridge and Lasso)	0.83 and 0.76 for Ridge and Lasso, respectively	^63^
Lasso regression			
mProcrustes	Linear regression model, which utilizes co-expressed genes as a model feature (for detailed explanation of co-expressed genes, see Multigene models section under Methods)	0.85, highest among CCC values for all batch correction methods used in benchmarking	N/A
Original batch	Expression data before the application of any batch correction techniques	0.58	N/A

To effectively analyze the data, we first sought to eliminate transcripts that would affect normalization due to inherent technical limitations or biological stochastic noise, particularly in cases where samples were sequenced using different protocols^17^. In total, 20,062 genes (AG - all genes) were used for TPM normalization and expression quantification in FFPE and poly-A RNA-seq data. We excluded 6,610 ( ~ 33%) genes that were not covered by one of the protocols. These included non-polyadenylated genes, genes not covered by V7 UTR probes, and genes that showed low expression levels (see Methods). Of the remaining 13,452 genes (AGEP: all genes after excluding problematic genes), 1438 ( ~ 10%) were grouped as cancer-specific, immune-related, and clinically relevant genes (BMGEP: biologically meaningful genes after excluding problematic genes; Table 2, Supplementary Data 7). Steps for projecting single samples onto the MET500 cohort using MNN and ComBat-Seq are described in Methods (Supplementary Fig. 3a). PCA projection plots for MNN-transformed (median CCC = 0.62) and ComBat-Seq-transformed (median CCC = 0.72) data showed that the transformed dataset does not perfectly fit the original poly-A expression profiles (Supplementary Fig. 3b, c). Particularly, the transformed data do not occupy the same area as either the EC-based or the poly-A RNA-seq holdout data on the PCA plots, showing the presence of pronounced batch effects even after data transformation by MNN and ComBat-Seq. The performance outcome for all selected batch correction methods are listed in Table 1 (Supplementary Fig. 3d).

**Table 2:** Gene groups selected for further analysis

Group name	Description^a^	Number of genes
AGs	All Genes	20,062
AGEP	All Genes, Excluding 6,610 Problematic Genes	13,452
BMG	Biologically Meaningful Genes	1,899
BMGEP	Biologically Meaningful Genes, Excluding Problematic Genes	1,438

^a^See Gene Filtering section in Methods for the definition of each group.

Taken together, our analysis of several existing batch effect correction methods shows that they cannot effectively transform EC-based FFPE RNA-seq data into poly-A-like RNA-seq gene expression profiles, as evidenced by the presence of pronounced batch effects even after data transformation. Therefore, we set out to develop a batch correction method that rectifies technical, protocol-specific batch effects in gene expression profiles between EC-based and poly-A RNA-seq.

### Development of Procrustes

**Fig. 2:**
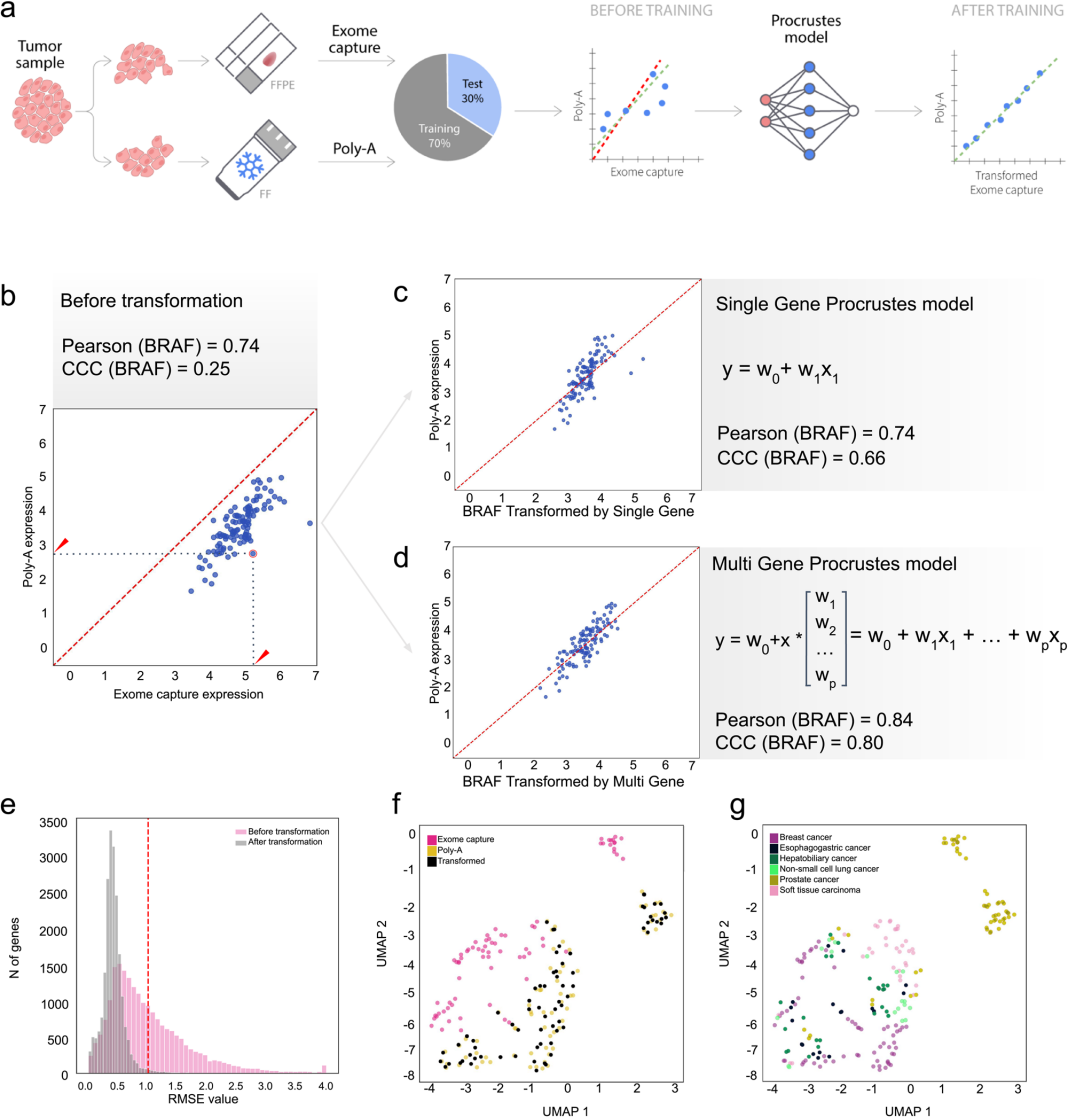
Development of Procrustes model. **a** Schematic showing Procrustes model development using FF and FFPE replicates. **b** Pearson correlation coefficient of BRAF expression between EC and poly-A-based RNA-seq. **c** Transformation of BRAF expression from EC- to poly-A-like after applying single gene Procrustes model and (**d**) multi gene Procrustes model. **e** Within-gene root mean squared error (RMSE) before and after EC RNA-seq data transformation on the MET500 dataset. **f** UMAP projections for top six cancer types present in the MET500 holdout set (Poly-A, EC, and transformed-EC). **g** UMAP projections for top six cancer types present in the MET500 holdout set; cancer-specific grouping retained (Poly-A, EC, and transformed-EC).

Here, we introduce Procrustes, a machine learning-based algorithm for batch correction (Fig. 2a). Based on the observed linear correlation between the expression of individual genes^18^ and the co-expression of specific genes (Supplementary Fig. 4; see Methods) in whole transcriptome data from different protocols, we developed two regression models. The first utilizes a single-gene regression model (sProcrustes), while the second uses a multi-gene regression model (mProcrustes) for cross-platform-derived RNA-seq data batch effect correction (see Methods). The sProcrustes model was developed based on the linear relationship of gene expression profiles between protocols, while the mProcrustes model was trained using the expression profile of each gene together with the topmost co-expressed genes in each protocol (see Methods). After selection of co-expressed genes, the final unified list contained anywhere from 1 to 56 genes, depending on the cancer type (see Methods, Supplementary Data 8). ElasticNetCV regression model^18^ was utilized to automatically adjust parameters with a three-fold cross validation. Further, to develop robust models for training, testing, and validation, we used the TCGA (phs000178)^19^ pan-cancer and GTEx normal tissue datasets to assess if cancer type diversity would affect model performance. As shown in Supplementary Fig. 5, we identified tissue-specific gene sets within GTEx and TCGA data and used this information to select the cancer types to be used for developing Procrustes (Supplementary Data 9, 10; see Methods). This information was also used to divide samples in the MET500 cohort into training and holdout sets, stratified by cancer type, to ensure similar proportions of different cancer types in each subset (Supplementary Fig. 6a–c). This same strategy was used in the evaluation of existing batch correction methods described in the preceding section.

Next, we trained and validated sProcrustes and mProcrustes using the same training and holdout subsets of the MET500 cohort^14^ that were previously used to assess other batch effect correction algorithms (see Methods and Limitations of existing batch effect correction methods). Following training, we applied sProcrustes to transform the expression values from EC to poly-A-like in the holdout set. As before, we used CCC^11^ to measure whether the algorithm accurately overcame the batch effects between poly-A and EC sequencing methodologies. The CCC values for more than 1,500 genes from the BMG group were above 0.75, demonstrating robust performance of sProcrustes and its ability to combine data from different sequencing protocols. By comparison, mProcrustes showed better performance, with higher CCC values within individual genes analyzed and a slightly improved gene-wise CCC across the BMG (Supplementary Data 11). For example, as shown in Fig. 2b, the correlation of BRAF expression between the modified EC and poly-A protocol was not high (r = 0.74) and had a low CCC of 0.25. Applying sProcrustes and mProcrustes yielded *r* and CCC values of 0.74 and 0.66, and 0.84 and 0.80, respectively, for BRAF expression (Fig. 2c, d). Further, transformation of EC data using mProcrustes also reduced the median within-gene root mean squared error (RMSE) from 0.83 to 0.40 (Fig. 2e). UMAP projections for the top six cancer types in the MET500 holdout set showed transformation of EC data to a poly-A-like dataset, with samples clustered by cancer type and not by protocol (Fig. 2f, g).

### Benchmarking Procrustes against existing batch correction methods

**Fig. 3:**
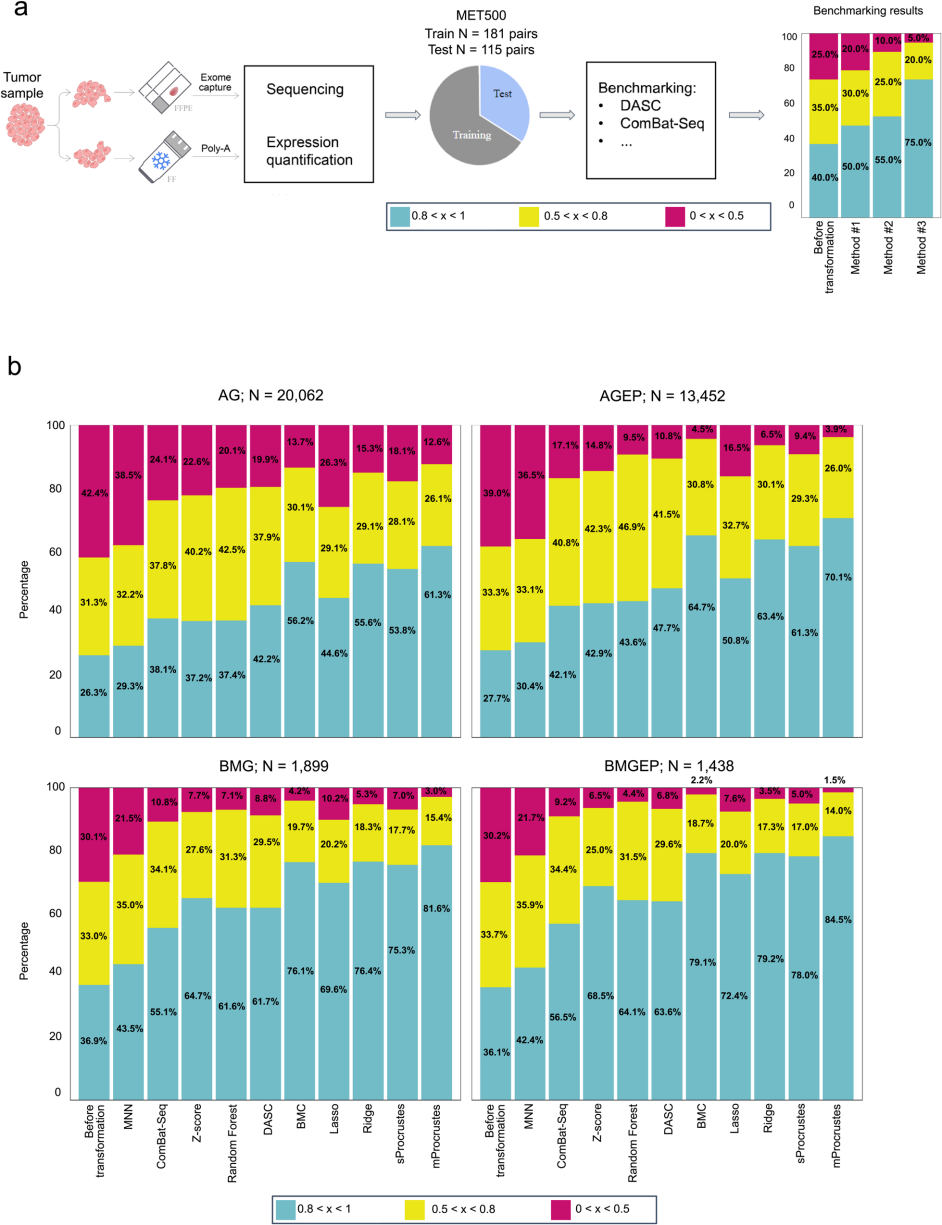
Benchmarking Procrustes’s performance. **a** Schematic showing our workflow for benchmarking Procrustes against other existing batch correction methods, as listed in Table 1. Batch correction was performed as described in Methods, and the outcome was measured based on CCC values. **b** Barplot comparing the performance of sProcrustes and mProcrustes against other batch correction methods in 4 predefined groups of genes (AG, AGEP, BMG, BMGEP) as measured by within-gene CCC values defined over 3 intervals (low [<0.5], medium [0.5–0.8], and high [>0.8]), as reflected by the color key included. All benchmarking was performed using the MET500 dataset (both EC-based and poly-A RNA-seq data). AG all genes, AGEP all genes excluding problematic genes, BMG biologically meaningful genes, BMGEP biologically meaningful genes excluding problematic genes.

We compared Procrustes to several existing batch correction algorithms, as listed and described in Table 1. Here, we also chose to use the MET500 dataset in our benchmarking approach because it is a comprehensive cancer cohort that encompasses samples of diverse diagnoses sequenced as replicates using poly-A RNA-seq and EC-based Agilent SureSelect V4 protocols. Thus, the use of the MET500 dataset enabled us to compare data generated by two different RNA-seq technologies within the same dataset (Fig. 3a).

Our analysis showed mProcrustes to consistently outperform all other batch effect correction algorithms selected for benchmarking, with higher CCC values across all four groups of gene sets described in Supplementary Data 6 (Fig. 3b, Supplementary Fig. 3d, Supplementary Data 12). Over 61% of genes (total *N* = 20,062) had a CCC higher than 0.8 across the two RNA-seq methodologies for each sample after correction. Moreover, approximately 84% of cancer-specific and microenvironment-related genes had a ССС higher than 0.8. Only the performance of Ridge regression, BMC, and sProcrustes came close to the performance of mProcrustes, with at least 75% of cancer-specific genes having a CCC higher than 0.8.

In addition, we assessed if transformation of EC data by mProcrustes affects gene signature analysis, a widely used application of GExP. Functional gene expression signature values (Fges) for previously reported gene signatures were calculated as described by Bagaev et al.^4^ using single sample gene set enrichment analysis (ssGSEA)^20^. As before, CCC was used to compare Poly-A and EC samples before and after correction. Our analysis showed the CCC values for most gene signatures (24 out of 29) before correction to be above 0.9. While these CCC values either increased or decreased slightly after correction (Supplementary Fig. 6a, b, Supplementary Data 13), the application of Procrustes did not alter the conclusion based on gene signature analysis. Taken together, these findings demonstrate the ability of mProcrustes to effectively analyze gene expression data obtained using different library preparation protocols.

### Evaluating the performance of Procrustes using sample set processed in-house

**Fig. 4:**
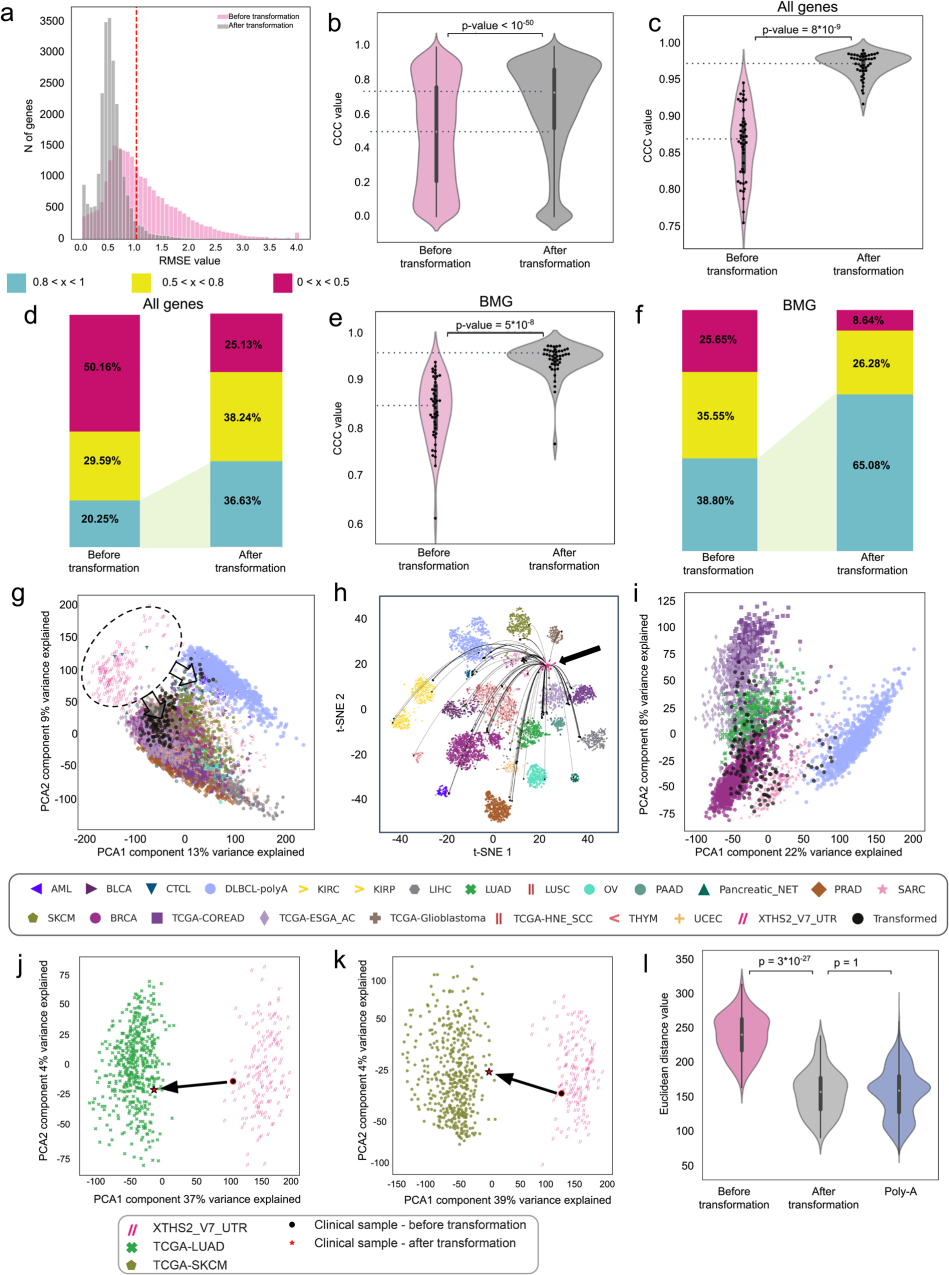
Assessing Procrustes’s performance on clinical samples. **a** Within-gene RMSE in clinical samples before and after correction by mProcrustes. **b** Violin plot showing within-gene ССС before and after correction by mProcrustes. The transformation method is depicted on the x-axis. The y-axis shows CCC values. For the nested box plots, whiskers indicate 25th percentile (bottom) and 75th percentile (top) +/− 1.5 IQR. **c** Violin plot showing ССС values for each EC/poly-A pair measured for all genes. The transformation method is depicted on the x-axis. The y-axis shows CCC values. For the nested box plots, whiskers indicate 25th percentile (bottom) and 75th percentile (top) +/− 1.5 IQR. **d** Performance of mProcrustes as measured by CCC for all genes (AG) present. Each color in the color key represents one CCC interval: 0<x < 0.5, 0.5<x < 0.8, and 0.8<x < 1.0. **e** Violin plot showing ССС values for each EC/poly-A pair using only the biologically meaningful gene (BMG) set. The transformation method is depicted on the x-axis. The y-axis shows CCC values. For the nested box plots, whiskers indicate 25th percentile (bottom) and 75th percentile (top) +/− 1.5 IQR. **f** Barplot showing performance of mProcrustes as assessed by CCC values for each EC/poly-A pair using only the biologically meaningful genes (BMG). The color key in (**d**) is used to reflect the same CCC intervals in (**f**). **g** PCA plot depicting batch effect between EC and TCGA cohort gene expression profiles. The transformed values (black) are projected onto their respective TCGA poly-A cohorts (colored). **h** t-SNE plots showing transformed values (black) and TCGA poly-A cohorts (colored). EC data form a separate batch. **i** PCA plot after applying mProcrustes showing the transformed values (black) for the six most prevalent cancer types included in the mapping and their corresponding TCGA cohorts (colored). **j** PCA plot depicting the projection of one clinical sample onto the TCGA-LUAD cohort. A set of 159 unpaired samples (pink) were processed in-house with XT HS2 V7 UTR. Transformation with Procrustes projected a single clinical sample from the EC library onto the TCGA-LUAD cohort, allowing comparison of gene expression between this clinical sample and a sample from the TCGA-LUAD cohort. **k** PCA plot depicting the projection of one clinical sample onto the TCGA-SKCM cohort. Transformation with Procrustes projected a single clinical sample from the EC library onto the TCGA-SKCM cohort, allowing comparison of gene expression between this clinical sample and a sample from the TCGA-SKCM cohort. Both (**j**) and (**k**) use the same color/pattern key included. **l** Violin plot showing Euclidean distances to PCA-centroids of nearest TCGA-cohorts. For the nested box plots, whiskers indicate 25th percentile (bottom) and 75th percentile (top) +/− 1.5 IQR.

To assess Procrustes’s clinical utility, we generated poly-A libraries from FF samples and XTHS2 V7 UTR libraries from FF or FFPE blocks for 129 sample pairs (Supplementary Fig. 6c, Supplementary Data 14). Sample pairs were split into training (*N* = 85 pairs) and test (*N* = 44 pairs) datasets (Supplementary Fig. 7a–d; see Methods). We tested the ability of mProcrustes to convert EC expression profiles into poly-A expression profiles. Median RMSE values decreased from 0.96 to 0.51 after transformation, which is comparable to variations typically associated with RNA-seq^17^ (Fig. 4a). The median CCC after transformation increased from 0.5 to 0.72 on a gene level (*N* of genes = 20,062; Fig. 4b). We also tested model performance characteristics in the 4 gene groups (Supplementary Data 7). In the AG group, for a pairwise comparison between EC and poly-A samples, median CCC after transformation increased from 0.87 to 0.97 (Fig. 4c) while the percentage of genes with a high CCC (>0.8) increased from 20% to 36% (Fig. 4d). The same comparison in the BMG group showed median CCC values to increase from 0.87 to 0.96 (Fig. 4e) and the percentage of genes with a high CCC (>0.8) to increase from 39% to 65% (Fig. 4f). A similar trend was observed for the other two groups of genes (Supplementary Fig. 7e–h).

Next, we assessed if the transformed EC samples were biologically comparable to poly-A sequenced cohorts. To do this, we used a separate batch of 159 clinical samples processed using our improved EC-based protocol (Supplementary Fig. 8a). As shown in Fig. 4g, h, PCA projection and tSNE plots of samples sequenced with the modified EC protocol formed a separate batch from all poly-A sequenced cohorts before transformation. This batch effect was removed upon applying mProcrustes (Fig. 4g–i). Finally, we mapped each of these 159 clinical samples onto their corresponding TCGA cohorts (Supplementary Data 15) and assessed batch effects before and after transformation. Our analysis showed high concordance between these 159 clinical lab samples and TCGA data after transformation (Supplementary Fig. 8b, c), as evidenced by a decreased median Euclidean distance to PCA centroid of the corresponding TCGA cohort (*p* < 1 ×10^−6^, Wilcoxon test; Fig. 4g–l).

To demonstrate the utility of Procrustes even without the wet lab improvement, we compared data from samples prepared with the unmodified EC-based (Agilent XT HS2 V7), the modified EC-based (Agilent XT HS2 V7 UTR), and the poly-A RNA-seq protocols, before and after transformation by mProcrustes. While the samples used here are from the same batch used for Supplementary Fig. 1a–c, only samples that had not been used previously for training Procrustes were used for this comparison.

As shown in Supplementary Fig. 8d–f, wet lab improvements on the existing Agilent XT HS2 V7 protocol alone were not sufficient in rectifying the batch effects. These batch effects were minimized after the application of Procrustes. Moreover, the Procrustes-transformed V7 data, while conforming better to the poly-A data (median CCC between non-modified V7 and poly-A data is 0.83 and 0.94 before and after transformation with mProcrustes, respectively, *p* < 2 ×10^−6^, Wilcoxon test), still fell short compared to the Procrustes-transformed V7 UTR data (median CCC between V7 UTR and poly-A data is 0.88 and 0.96 before and after transformation with mProcrustes, respectively, *p* < 2 ×10^−6^, Wilcoxon test; Supplementary Fig. 8f). As such, our analysis revealed a performance gradient, where Procrustes-transformed V7 UTR data aligned best with poly-A data, followed by Procrustes-transformed V7 data. Non-transformed data performed the worst. These observations indicate that Procrustes is needed to transform gene expression data to correct and subsequently minimize batch effects when comparing data obtained from different library preparation protocols. This is especially important in the analysis of clinical samples in reference to public databases, where the analysis outcomes are often used to guide treatment decisions.

### Clinical utility and limitations

Our testing of Procrustes also revealed some of its limitations. Specifically, we show some examples of genes that may be challenging for Procrustes to transform and compare with poly-A RNA-seq data (Supplementary Fig. 4e). These examples are *GNRHR*, *FGF4*, and *FGF6*, which have all been shown to play important roles in cancer biology^21–23^. As shown in Supplementary Fig. 4f, poly-A RNA-seq data for the TCGA cohorts show *GNRHR* expression to be undetectable. The same is true for poly-A samples from the MET500 cohort (Supplementary Fig. 4e). Therefore, we conclude that it is not a database-specific artifact. Importantly, this limitation is unlikely to be overcome by any batch correction method at this time. For *FGF4* and *FGF6*, there are limited data points for their expression beyond zero at this time, thus limiting their use for training and testing Procrustes (Supplementary Fig. 6e). These limitations and how we propose to overcome them are discussed in detail in the Discussion section.

While evaluating other existing batch correction methods and benchmarking Procrustes against these methods using the MET500 dataset, we showed that Procrustes can transform gene expression data obtained using the Agilent SureSelect V4 protocol and improve their alignment with the poly-A RNA-seq data for the same samples (Fig. 2e–g). Here, we further demonstrate that Procrustes can even transform data obtained using the Agilent XT HS2 V7 protocol as-is without any protocol modification, to achieve some improvement in alignment with poly-A data. This is an important advantage of Procrustes, particularly in clinical settings where protocol modification is not always feasible.

Moreover, unlike the existing batch correction methods included in our benchmarking target list (Table 1), Procrustes can be used directly without any modification to compare a single, separate sample (or a small number of samples) to samples in a large poly-A cohort. As a simple linear regression model, Procrustes can be easily and efficiently applied to a single sample or a small number of samples. Figure 4j, k demonstrate the projection of a single clinical sample onto a specific TCGA cohort after data transformation by Procrustes. In these plots, a single sample, each from a library of advanced lung adenocarcinoma (LUAD) clinical samples and skin cutaneous melanoma (SKCM) clinical samples, respectively, were simply projected onto their respective TCGA cohorts. More importantly, Procrustes could do this without the need for the complicated steps described for MNN and ComBat-Seq (see Methods). This is another valuable advantage that Procrustes has over other batch correction methods, and one that raises its clinical applicability.

Taken together, our analysis demonstrates the ability of mProcrustes to transform data from clinical FFPE samples processed using an EC-based RNA-seq protocol, thus enabling integration with RNA-seq data generated from FF samples within the TCGA dataset or other gene expression cohorts processed with a poly-A-based library preparation technique. In this study, we further demonstrated the ability of Procrustes to transform EC-based data obtained using the preexisting Agilent Sureselect V4 and Agilent XT HS2 V7 protocols, a feature that underscores its utility in the clinics.

## Discussion

In the last decade, RNA-seq has played a crucial role in shaping our understanding of the functional genome^24^. Poly-A RNA-seq has seen widespread adoption in research as it provides a comprehensive overview of the transcriptomic landscape. However, this protocol has seen limited use in the clinical setting where samples are routinely stored as FFPE specimens, causing the input RNA to be highly degraded with partial 3’ ends. EC-based methods overcome this limitation, but still fail to recapitulate poly-A RNA-seq data. In this study, we optimized an EC-based RNA-seq protocol^2,24^ to recapitulate poly-A RNA-seq data starting from FFPE samples, allowing a wealth of transcriptomic data to be collected from clinical samples and compared to data obtained using the more conventional poly-A sequencing protocol. This optimized protocol included probes to target the untranslated regions of all protein-coding mRNAs. In doing so however, we observed marked batch effects when comparing FF sample-derived poly-A RNA-seq data to both FF and FFPE sample-derived data that likely arose due to differences in protocols used to generate the data.

To overcome this remaining limitation computationally, we developed Procrustes, a linear regression-based model to transform EC-based data to poly-A RNA-seq data, that can rectify batch effects stemming from differences in library preparation protocols. We developed and benchmarked two different models (sProcrustes and mProcrustes) to demonstrate the ability of our algorithm to integrate and compare datasets from large cohorts sequenced using different methods. We believe that Procrustes is a revolutionary method for projecting gene expression data from a single sample onto a larger cohort of RNA-seq data, thus allowing analysis and comparison of RNA-seq data across different sample preparation protocols in clinical settings. In this regard, we also showed that Procrustes can transform gene expression data obtained using the Agilent SureSelect V4 and Agilent XT HS2 V7 protocols without the need for complicated data-processing steps and protocol modification. This is an important and invaluable advantage of Procrustes over other existing batch correction methods, especially in the clinical setting.

Moreover, utilization of linear regression ensures high interpretability for transformed gene expression values, which makes it valuable in the clinical context. For instance, upon receiving EC-based gene expression values for a sample from a lung cancer patient, a user can simply apply Procrustes to project this particular lung cancer sample onto the appropriate publicly available poly-A cohort (e.g., TCGA-LUSC or TCGA-LUAD). Thus, we envision Procrustes as a batch correction tool for EC-processed samples for comparison with publicly available poly-A cohorts.

Nonetheless, linear regression models suffer from limited sample sizes wherein the estimated coefficients of the model can become large, making the model sensitive to inputs and possibly unstable. Our study utilized a limited sample set (*n*) that exceeded the number of input predictors (*p*) or variables (gene features in this study), giving rise to the so-called *p » n problem*, which can result in a model that will fit to noise instead of generalization. One approach to addressing this issue is to use a regularization method and modify the loss function to include additional penalty costs for a model that has large coefficients. In this study, we used ElasticNet regression models that penalize noisy features (genes) to minimize the size of all coefficients, resulting in better model performance.

By defining and identifying co-expressed genes to be used for the mProcrustes model, we demonstrated that mProcrustes can robustly correct for differences between poly-A and EC-based data. Validation of mProcrustes on multiple datasets revealed high concordance between the gene expression profiles produced by the poly-A RNA-seq and EC-based protocols after application of mProcrustes. We also demonstrated that mProcrustes can handle complex batch effects without negatively impacting GExP studies such as developing predictive gene expression signatures from transcriptomic data. We also showed in simulations that mProcrustes generally not only outperforms other methods in removing technical batch effects, but also preserves the ability to predict functional gene signatures (Supplementary Fig. 7a, b), showing its potential to compare biological signals regardless of the sequencing technology used for data generation.

It is important to note that Procrustes was created specifically to address batch effects associated with EC-based protocols when applied to FFPE samples. In particular, Procrustes performs best when comparing data from EC-based V4, V7, and V7 UTR protocols to poly-A RNA-seq data, as described in this study. Since Procrustes was trained to compare RNA-seq data across EC-based and poly-A RNA-seq protocols, we do not anticipate it to perform well in comparing data generated by other RNA-seq protocols (such as total RNA-seq or other EC-protocols not included in this study) with data from poly-A RNA-seq. This is because the relationship between other types of RNA-seq protocols and the poly-A RNA-seq protocol likely differs from the relationship between EC-based and poly-A RNA-seq protocols. Moreover, the expression pattern for the same gene may differ depending on the protocol used to obtain the data. For instance, while V4 could detect *GNRHR* expression in the MET500 cohort, poly-A RNA-seq data for both the MET500 and TCGA cohorts show *GNRHR* expression to be undetectable. Addressing these different relationships and discrepancies will most likely require: 1) users to train their own model using either the training and test paired poly-A-EC data reported in this study, or different datasets that correspond to different protocol pairs of their interest; or 2) the use of different methodologies and modeling approaches than the ones described in this study, thus necessitating the creation of an algorithm that is, at least in part, different from Procrustes. The latter is an avenue we plan to explore in future iterations of Procrustes or other new algorithms.

Current limitations of this study include a limited sample size. Small sample sizes can lead to a narrow distribution of gene expression values for particular genes, exacerbating the issue with the number of model features (*p*) being greater than the number of samples (*N*) (*p*»*n* issue), especially for mProcrustes. Future iterations of Procrustes will consider incorporation of data from additional cancer types with larger sampling to create more robust gene sets for analysis. Another limitation in this study is the choice of filtering criteria to define and remove noisy transcripts wherein we excluded all non-expressing poly-A genes from gene sets to train mProcrustes. These limitations may pose some challenges for Procrustes to transform the expression data for some genes, as shown in Supplementary Fig. 4e, f. Improvements will require better definitions of features for model training and validation. Possible strategies to address these limitations include expanding the sample size to include more samples with expression values greater than zero, or expanding the EC-based cohorts in order to obtain a more accurate picture of the expression levels of genes of interest. Future work is also needed to better define co-expressed cancer-specific gene sets for model development. While we filter noisy transcripts before applying Procrustes, the current iteration of Procrustes cannot correct the data for every gene that remains. Our future work will involve employing more sophisticated approaches to increase model performance. Some examples include better definition of gene relationships between protocols, additional feature preprocessing, and improved model hyperparameter tuning as described by Sabourin et al.^25^ and by Feng and Yu^26^.

In genomic sequencing, the utility of batch correction tools extends beyond the method employed in this study, potentially benefiting other methods as well. For example, approaches like ATAC-seq, ChIP-seq, and DNase-seq might also leverage such tools, given their susceptibility to batch effects^27,28^. However, when compared to bulk RNA-Seq where we found library preparation to be the predominant source of batch variability that we could effectively address with Procrustes, these techniques present additional complexities. For different sequencing approaches, some confounding factors^28–31^ may contribute more to batch effect than library preparation methods. The multifactorial nature of batch effects makes the straightforward application of Procrustes to these techniques less certain. Instead, it necessitates a more in-depth investigation and possibly the development of tailored correction strategies. Such an approach is crucial for accurately understanding and mitigating the unique sources of variability, ensuring the reliability and reproducibility of results despite the diversity and variation in genomic sequencing technologies.

We believe the development of our optimized EC-based RNA-seq protocol and Procrustes will provide a workflow that will enable cross-platform analysis of heterogeneous transcriptome databases between different protocols. Further, our approach now allows for the accurate projection of a single patient’s transcriptomic data to larger, diagnosis-matched cohorts and will accelerate the adoption of whole transcriptome sequencing in the clinic for diagnosis and therapeutic intervention.

## Methods

### Specimen procurement

All research specimens in this study were obtained from commercially available sources. Fresh frozen (FF) and formalin-fixed paraffin-embedded (FFPE) samples were purchased from commercial sources, including Cureline Tissue Bank (Brisbane, CA), Accio Biobank Online (Newmarket, Suffolk, United Kingdom), UMass Medical Memorial Biospecimen and Tissue Bank (North Worcester, MA), and OriGene (Rockville, MD). Cell Lines listed in Supplementary Data 3 were purchased from American Type Culture Collection (ATCC, Manassass, VA) except GM12877 and GM12878, which were purchased from Coriell Institute for Medical Research (Camden, NJ) (Supplementary Data 3). According to ATCC, mycoplasma is not detected in the cell lines we procured. For cell lines procured from Coriell Institute for Medical Research, information concerning mycoplasma contamination is not available, and no further testing was performed in our laboratory. All cell lines were used to compare gene expression levels as obtained by different RNA-seq protocols (technical comparison). No biological conclusions were derived from these findings. Sorted cell populations were derived from healthy donors from whole blood from Research Blood Components^17^ (Watertown, MA). Clinical samples (*N* = 74) were also included in this study (Supplementary Data 14). Each patient provided informed consent. The use of clinical samples was conducted in accordance with the Declaration of Helsinki and has been granted exemption from ethics approval by the Biomedical Research Alliance of New York (BRANY) Institutional Review Board (IRB) (BRANY study #22-12-938-853).

### FFPE sample processing

FF tissues were stored at −80 °C until they were processed. Tissue samples were transported to a cryostat pre-chilled to −20 °C and divided in half using a razor blade, maintaining representative and morphologically similar parts of the tissue for both halves. Half of each FF specimen was prepared as an FFPE block, and the other half was used directly for downstream RNA extraction. Prior to FFPE processing, tissue specimens were cut into 5-micron sections. For FFPE processing, samples were placed into embedding cassettes, which were directly immersed into 10% neutral buffered formalin and incubated on an orbital shaker for 24 h at room temperature. Cell lines were grown in appropriate media and density according to ATCC or Corielle guidelines. Agarose cushions were made from 0.2 ml of 2% agarose in PBS in 1.5-mL centrifuge tubes. Fresh cells were placed onto solidified agarose cushions and spun down in a centrifuge at 2,000 g for 8 min. The supernatant was removed and replaced with 10% neutral buffered formalin for 24 h at room temperature. Tissues were then processed on a tissue processor machine (Leica TP1020) undergoing two 30-min cycles each of 70%, 95%, and 100% ethanol, two 30-min cycles of xylene, and two 1-h cycles of paraffin. Post-processing, the tissues were embedded into paraffin blocks using the HistoCore Arcadia Embedding Center (Leica).

### FF sample processing

FF tissue specimens were stored at −80 °C until RNA extraction was performed. For sorted cell populations from blood, cells were placed into a homogenization buffer (Promega, Maxwell SimplyCells RNA) and stored at −80 °C until RNA extraction. Sorted cell populations from whole blood were obtained from the peripheral blood of healthy donors procured from Research Blood Components (Watertown, MA)^17^. Briefly, peripheral blood mononuclear cells were prepared, labeled with monoclonal antibodies, and sorted with a BD FACSAria III through a 100 mm nozzle^17^.

### RNA extraction

RNA was extracted from FF tissue specimens using the Qiagen AllPrep DNA/RNA Kit (catalog# 80284). RNA from FF cell line pellets was extracted using the Maxwell RSC simplyRNA Cells kit (catalog# AS1390). FFPE specimens were cut into 10-µm thick sections and RNA was extracted using either the Qiagen AllPrep DNA/RNA FFPE kit (catalog# 80234) or Maxwell RSC FFPE RNA (catalog# AS1440) extraction kit. All extractions were performed as per the manufacturer’s instructions.

### Library preparation and sequencing

Poly-A libraries were generated using the Illumina Truseq Stranded mRNA kit following the manufacturer’s recommended protocols (catalog# 20040532). Exome capture sequencing libraries were generated using the Agilent SureSelect XT HS2 RNA kit (catalog# G9993B) according to the manufacturer’s recommended protocol. In this study, two different probe sets were utilized for the hybridization procedures: SureSelect Human All Exon V7 (Agilent, catalog# 5191-4029) and a custom probe set, V7 UTR, designed to cover all the coding exons and 5‘ and 3‘ UTR regions (Supplementary File 1). All RNA libraries were sequenced on an Illumina NovaSeq 6000 sequencer. Samples were sequenced to a median target coverage of ~54 M reads, with 151 bp paired-end sequencing. V7 UTR probe coverage was assessed using GitHub - openvax/gtfparse: Parsing tools for GTF (gene transfer format) files^32^ and BEDTools^33^ (Supplementary Fig. 1b).

### NGS data preprocessing and quality control

FastQC v0.11.5^34^, FastQ Screen v0.11.1^35^, RSeQC v3.0.0^35,36^, and MultiQC v1.6^37^ were used to perform quality control (QC) of all NGS samples. Sample correspondence was confirmed by HLA comparison using OptiType^38^ for RNA-Seq. RNA-seq reads were aligned to GRCh38.d1.vd1 using Kallisto v0.42.4^39^ and normalized into transcripts per million (TPM). TPM values were log2-transformed with an addition of 1 before model development and data analysis.

### Data visualization

Data visualization was performed using matplotlib (v1.5.1)^40^ and seaborn (v0.7.1) for Python^41^. UMAP^42^ and tSNE^43^ were used to visualize cancer types clusters.

### Statistics and reproducibility

Wilcoxon signed-rank test was used to assess the difference between samples (pairwise) before and after transformation by the Procrustes models. Pearson and Spearman correlations were calculated to define co-expressed genes. All statistical tests were performed using the SciPy Python library^44^. Post hoc statistical power analysis was performed using the “FTestAnovaPower” function from the statsmodels Python library. A standardized mean difference approach was used for effect size calculation, which resulted in an effect size value of 0.56 for concordance correlation coefficient before and after application of Procrustes. For alpha=0.0001 and power=0.95, a value of 108 for optimal sample size was obtained. Our final sample size of 129 samples processed in-house exceeds this value. Arithmetic mean, median, and standard deviation (STD) were calculated using NumPy. Root mean squared error (RMSE) was calculated for data before and after transformation (within gene) using the scikit-learn Python library^45^. Concordance correlation coefficients (CCCs) were calculated according to Lawrence I-Kuei Lin^11^.

To explore batch effects, we utilized technical replicates prepared with either EC-based or poly-A-based library preparation methods from either FFPE or FF tissues.

### Data decomposition

Principal component analysis (PCA) was performed with randomized singular value decomposition (SVD)^46^ using PCA decomposition from scikit-learn^18^. For decomposition, the mutual nearest neighbors (MNN) method (GitHub - chriscainx/mnnpy: An implementation of MNN (Mutual Nearest Neighbors) correct in python)^47^ was used^13^.

### Comparison of batch correction methods: MNN-based batch correction, ComBat-Seq, DASC, Z-score normalization, and batch mean centering

Each paragraph below describes the procedure used to compare one batch correction method to Procrustes. The performance of each method was evaluated by calculating CCC for the expression values before and after correction (Table 1).

MNN: Because mutual nearest neighbors (MNN)^13^ cannot be used to project a single, separate sample from one batch to another, each sample from the holdout-EC set was progressively added to the training EC set (Supplementary Fig. 3a). The MNN batch correction algorithm was then applied to the resulting EC set and the Train-poly-A set. These steps were performed with each of the samples from holdout-EC set separately. Next, we compared the full MNN-transformed set of samples with the holdout poly-A cohort.

ComBat-Seq: Given that ComBat-Seq^48^ cannot be applied to individual samples without additional steps, we used the same approach previously utilized with the MNN-algorithm. As it is required by the ComBat-Seq manual, we applied it to raw gene expression counts from the poly-A-holdout and EC-holdout MET500 subsets without prior TPM normalization. Next, values acquired after ComBat-Seq transformation were TPM-normalized. CCC was calculated between resulting values and original values of the poly-A-holdout MET500 subset.

DASC: To test whether DASC^49^ (Supplementary Note 2) could be used to correct batch effects between poly-A and EC-based RNA-seq data, we applied it to both poly-A and EC training MET500 subsets, which resulted in matrix of coefficients with a shape (*n*, 2), where *n* is a number of genes (*N* = 20,062).

$$\left[\begin{array}{cc}{{{{{{\rm{m}}}}}}}_{1} & {{{{{{\rm{d}}}}}}}_{1}\\ {{{{{{\rm{m}}}}}}}_{2} & {{{{{{\rm{d}}}}}}}_{2}\\ \cdots & \cdots \\ {{{{{{\rm{m}}}}}}}_{{{{{{\rm{n}}}}}}} & {{{{{{\rm{d}}}}}}}_{{{{{{\rm{n}}}}}}}\end{array}\right]$$

Next, using this matrix of coefficients, we transformed gene expression values for each gene in the EC-holdout MET500 subset using the following formula:

${Transformed\_ \, Ex}{p}_{g}={Ex}{p}_{g}\times {m}_{g}\div{d}_{g}$, where ${Ex}{p}_{g}$ is the expression of gene $g$ in the EC-holdout subset from the MET500 cohort, and ${m}_{g}$ and ${d}_{g}$ are coefficients from the matrix resulting from applying DASC to the EC and poly-A MET500 training subset. After that, for ${Transformed\_Exp}$, all values below zero were equalized to zero, whereas gene values higher than 3 $\sigma$ from mean were clipped to mean plus 3 $\sigma$. Finally, the resulting ${Transformed\_ \, Exp}$ was used to calculate CCC for the poly-A holdout subset of the MET500 cohort.

Z-score: Usually, Z-score values are used by themselves to nullify batch effect in downstream analysis, and calculated using this formula:

$Z=\frac{\chi -\mu }{\sigma }$, where $\chi$ corresponds to gene expression in current sample, $\mu$ corresponds to gene expression mean, and $\sigma$ corresponds to gene expression STD.

Since our main purpose of applying batch correction methods was to transform original EC values into poly-A-like values for a single sample, we transformed data from the EC-holdout subset using the following steps. First, we calculated gene expression mean and STD for both the EC and poly-A training MET500 subsets. Next, we calculated Z-scores for the EC-holdout subset based on the mean and STD from the previous step:

${Z}_{{EC}-{holdout}}=\frac{{\chi }_{{EC}-{holdout}}-{\mu }_{{EC}-{training}}}{{\sigma }_{{EC}-{training}}}$, where ${\chi }_{{EC}-{holdout}}$ is gene expression of EC-holdout MET500 subset, and ${\mu }_{{EC}-{training}}$ and ${\sigma }_{{EC}-{training}}$ are gene expression mean and STD of MET500 training subset, respectively. Finally, we acquired transformed expression for the EC-holdout MET500 subset using this formula:

${X}_{{EC}-{transformed}}={Z}_{{EC}-{holdout}}\times {\sigma }_{{polyA}-{training}}+{\mu }_{{polyA}-{training}}$, where ${\sigma }_{{polyA}-{training}}$ and ${\mu }_{{polyA}-{training}}$ are STD and mean values for the poly-A-training MET500 subset, respectively.

Batch mean centering (BMC) normalization: To perform batch correction with BMC normalization, we calculated the mean gene expression value for each gene for both EC and poly-A RNA-seq data in the training subset of the MET500 cohort. Following the same logic as for Z-score batch correction, we performed BMC normalization by subtracting EC-based means and adding poly-A-based means to samples from the EC-holdout subset of the MET500 cohort:

${X}_{{EC}-{transformed}}={X}_{{EC}-{holdout}}-{\mu }_{{EC}-{training}}+{\mu }_{{polyA}-{training}}$, where ${X}_{{EC}-{holdout}}$ corresponds to gene expression in EC-holdout MET500 subset, and ${\mu }_{{EC}-{training}}$ and ${\mu }_{{polyA}-{training}}$ are mean gene expression values for training EC and poly-A MET500 subsets, respectively. The resulting poly-A-like values were used to assess CCC in comparison to the original data of the poly-A-holdout subset of the MET500 cohort.

### Gene filtering

In order to ensure that data from different sequencing protocols were more comparable, all transcripts of non-coding biological types were excluded before TPM normalization as previously performed in the TCGA mRNA analysis pipeline for FPKM^50^. Histone-coding and mitochondrial gene transcripts were also excluded due to uneven enrichment with different RNA extraction methods, e.g., poly-A vs total RNA^51^. The resulting set of genes was retained for TPM normalization and expression quantification and contained 20,062 genes, including cancer-specific, immune-related, and clinically relevant genes (*N* = 1899). For each transcript remaining after this pre-filtering step, the effective length was found as the sum of exon lengths intersected with the Covered.bed (Supplementary File 1) file with merged probes and sequences with a minimum of 95% homology. Then, effective lengths of transcripts were summed up within each gene, and the same was done for actual transcript lengths. Sums of effective transcript lengths were divided over sums of actual transcript lengths within each gene. The resulting fraction is depicted on the x-axis of a histogram (Supplementary Fig. 1b). Genes with a fraction less than 0.5 were considered problematic.

### Tissue-specific expression

Tissue-specific expression values were analyzed on GTEX and TCGA cohorts (Supplementary Data 15). We selected cohorts that had more than 50 cancer and 20 normal samples. Gene expression was TPM-normalized, log2-transformed, and scaled from 0 to 1 for GTEX and TCGA separately. The tissue-specific expressed genes were collected for each of the selected GTEX, TCGA-tumor, and TCGA-normal cohorts. Genes were considered tissue-specific when the median of scaled expression was at least 0.5 in the selected cohorts and less than 0.2 in the remaining cohorts. All genes from GTEX, TCGA-tumor, and TCGA-normal were combined to form a final list of 4,960 genes.

### TCGA mapping

A total of 159 non-paired XTHS2 V7 UTR samples were selected by QC for further transformation by modeling and mapping onto poly-A cohorts (Supplementary Data 15). Two public cohorts (AML [phs001657.v1.p1]^52,53^, PNET [GSE98894]^54,55^), one internal cohort (CTCL; restricted use), and 19 TCGA cohorts were utilized to create a single poly-A RNA-seq cohort (Supplementary Data 16). SVD was performed using randomized SVD on the unified poly-A cohort^46^ to obtain 4,000 principal components (PCs) (Supplementary Fig. 9a, b). For each of the 22 poly-A subset cohorts, centroids were identified using the NearestCentroid method^18^. Next, each of 159 EC samples was mapped to the nearest centroid of the corresponding cohort before and after transformation by Procrustes. Additionally, the closest cohort to each sample was defined by lowest Euclidean distance. The resulting cohort was considered as a predicted cohort and compared to the cohort class, reflecting sample diagnosis. The intersection of expected and resulting cohorts was calculated as a percentage of the overlap.

### Development of linear models for Procrustes

Given $p$ predictors, the common linear regression model predicts the response ($y$) using the following formula:

$$y={w}_{0}+{w}_{1}{x}_{1}+...+{w}_{p}{x}_{p}$$

A model fitting procedure produces a vector of $w$ coefficients. For example, the ordinary least squares (OLS) estimates are obtained by minimizing the residual sum of squares. However, OLS often performs poorly for prediction and interpretation. Penalization techniques are utilized to improve OLS estimations. The Lasso and Ridge regressions are penalized least squares methods imposing l1- and l2-penalties on the regression coefficients, respectively^56^. For expression data projection from one sequencing protocol to another, $y$ is the projected expression, and $x$ is a vector of predictors. When comparing cross-platform gene expression levels, where most gene expression profiles show a linear dependence between platforms, we used a simple linear regression model with the equation:

$$y={w}_{0}+{w}_{1}{x}_{1}$$

where x_1_ is the target gene expression in EC and $y$ is its projection to poly-A.

We used the widely accepted machine learning tool *ElasticNet*, which is based on regularization of linear regression coefficients by adjusting both l1- and l2-penalties^56^ by minimizing the following equation:

$$\frac{1}{2{n}_{{samples}}}{{{\Vert }}X\omega -y{{\Vert }}}_{2}^{2}+\alpha \rho {{{\Vert }}\omega {{\Vert }}}_{1}+\frac{\alpha \left(1-\rho \right)}{2}{{{\Vert }}\omega {{\Vert }}}_{2}^{2}$$

where α is a constant which multiplies l1- and l2-penalties; $p$ is an l1-ratio ranging from 0 to 1, where a value equal to 1 indicates the use of the lasso penalty alone. Further, we used the ElasticNetCV (scikit-learn, Python) version^18^, which provides an internal cross-validation estimator. The cross-validation estimator is capable of specified model parameter searches (i.e. α and l1-ratio) with more computing power efficiency compared to canonical estimators^42^.

### Multigene models

Correlations between genes are utilized in algorithms of recovering missed values of gene expression^57^. Methods such as the Weighted Gene Co-Expression Network Analysis (WGCNA) calculate a connectivity score/measure between genes to external gene information^58^. This supports the possibility of using the expression of several genes in our linear projection of a target gene between EC and poly-A. We developed the mProcrustes model to utilize co-expressed genes wherein for each predictor *x*_1_,*x*_2_,...*x*_*p*_, there exists a corresponding vector of coefficients $w$ for each target gene, where predictors (or genes) for such a model can be selected based on Pearson correlation coefficients.

First, we selected 13 TCGA cancer-specific cohorts, each containing at least 50 samples. Then, the Pearson correlation coefficient was calculated for each gene in each cohort. For every gene in each of the chosen cohorts, we selected five genes with the highest correlation values (Pearson correlation of at least 0.7) to generate a summarized list of coexpressed genes for each gene. Next, these same steps were used to select the ten most correlated genes for each gene in the MET500-poly-A cohort to generate a similar summarized list. Finally, we merged those summarized lists into a finalized list of co-expressed genes for use with mProcrustes. The end result was a list of 19431 genes that had at least one co-expressed gene, up to a maximum of 56 co-expressed genes (Supplementary Fig. 9c).

### Ridge and Lasso regression

To test whether it is necessary to use a combination of l1-/l2-penalties or they can be used separately, we performed model training and validation in the same way as for sProcrustes. For both Ridge and Lasso, we made separate linear regression models for each gene using GridSearchCV cross-validation (see grid for hyperparameter tuning in Supplementary Note 1) on the training samples from the MET500 dataset. Next, we assessed CCC on the MET500 holdout subset.

### Random Forest regression

We used Random Forest Regression (RFR; RandomForestRegressor, scikit-learn^18^) to test whether non-linear methods might perform better than linear regression. We applied RFR to each gene separately on the MET500-training subset as was done for sProcrustes, Ridge regression, and Lasso regression. We used GridSearchCV to tune hyperparameters with cross-validation (see grid for hyperparameter tuning in Supplementary Note 1). Next, we assessed CCC on the MET500 validation subset.

### Reporting summary

Further information on research design is available in the Nature Portfolio Reporting Summary linked to this article.

### Supplementary information

Supplementary Information

Description of Supplementary Materials

Supplementary File

Supplementary Data 1-16

Reporting Summary

## Data Availability

Sequencing raw data used for model training and validation in this study can be accessed at the NCBI Short Read Archive (SRA) with accession number PRJNA1073545. Accessions for the datasets used in this study include the following: phs000178 (TCGA)^19^, phs000673.v2.p1 (MET500^14^), phs001657.v1.p1 (AML^52,53^), GSE98894 (PNET^54^). Source data are provided for this manuscript (https://zenodo.org/records/10552676). Some data from this study are not publicly available because it contains information that could compromise research participant privacy/consent.

## Code availability

All code is deposited online (https://github.com/BostonGene /Procrustes)^59^ and will be publicly available at the time of publication. Model weights for gene expression transformation and the calculated gene expressions for all samples included in the analysis for this manuscript are also available at this repository. The deposited code can be used to transform expression values from EC (Agilent V4, V7, and V7 UTR) into poly-A-like values in order to reproduce the CCC values derived from this study.
