# Supplementary material for: Procrustes is a machine-learning approach that removes cross-platform batch effects from clinical RNA sequencing data
Source: Commun Biol. 2024 Mar 30;7:392. doi: 10.1038/s42003-024-06020-z (PMC10981711; doi:10.1038/s42003-024-06020-z)
Supplement: Supplementary file 1 — Supplementary Information [file 42003_2024_6020_MOESM1_ESM.pdf]

Supplementary Note 1: Hyperparameter tuning

Scikit-learn python library was used to train *Random Forest Regression, Ridge, and Lasso* with hyperparameters tuning and cross-validation.

Function *GridSearchCV* was used with the following parameters:

cv	*ShuffleSplit(n_splits=5, test_size=0.33, random_state=42)*
scoring	*make_scorer(concordance_correlation_coefficient_scoring, greater_is_better=True)*
param_grid	**see below*

For hyperparameter tuning, the following parameters grid was used (as a *param_grid* parameter*)*:

Lasso<br>*Ridge*	*'alpha': [0.1, 0.15, 0.2, 0.25, 0.3, 0.35, 0.4, 0.45, 0.5, 0.55, 0.6, 0.65, 0.7, 0.75, 0.8, 0.85, 0.9]*<br>*'l1_ratio' : [.1, .5, .7, .9, .95, .99, 1]*
Random Forest Regression	*'n_estimators': [100, 200, 300]*<br>*'max_depth': [None, 2, 3, 4, 5, 10, 20]*<br>*'min_samples_split': [0.1, 0.2, 0.3]*<br>*'min_samples_leaf': [0.1, 0.2, 0.3]*

Supplementary Note 2: DASC installation

To reproduce results for DASC batch correction method we recommend following the steps below using R:

1) Installation:

```
### cvxclustr
url <- "https://cran.r-project.org/src/contrib/Archive/cvxclustr/cvxclustr_1.1.1.tar.gz"
pkgFile <- "cvxclustr_1.1.1.tar.gz"
download.file(url = url, destfile = pkgFile)
install.packages(pkgs=pkgFile, type="source", repos=NULL)
unlink(pkgFile)
library(cvxclustr)

### DASC
```

```
url <-
"https://bioconductor.statistik.tu-dortmund.de/packages/3.6/bioc/src/contrib/DASC_0.99.11.tar.gz"
pkgFile <- "DASC_0.99.11.tar.gz"
download.file(url = url, destfile = pkgFile)
install.packages(pkgs=pkgFile, type="source", repos=NULL)
unlink(pkgFile)
library(DASC)
```

2) Recommended virtual environment:

**Package**	**Version**
Biobase	2.60.0
BiocManager	1.30.21
cvxclustr	1.1.1
DASC	0.99.11
DescTools	0.99.49
doParallel	1.0.17
foreach	1.5.2
igraph	1.4.3
knitr	1.43
NMF	0.26
umap	0.2.10.0
vioplot	0.4.0

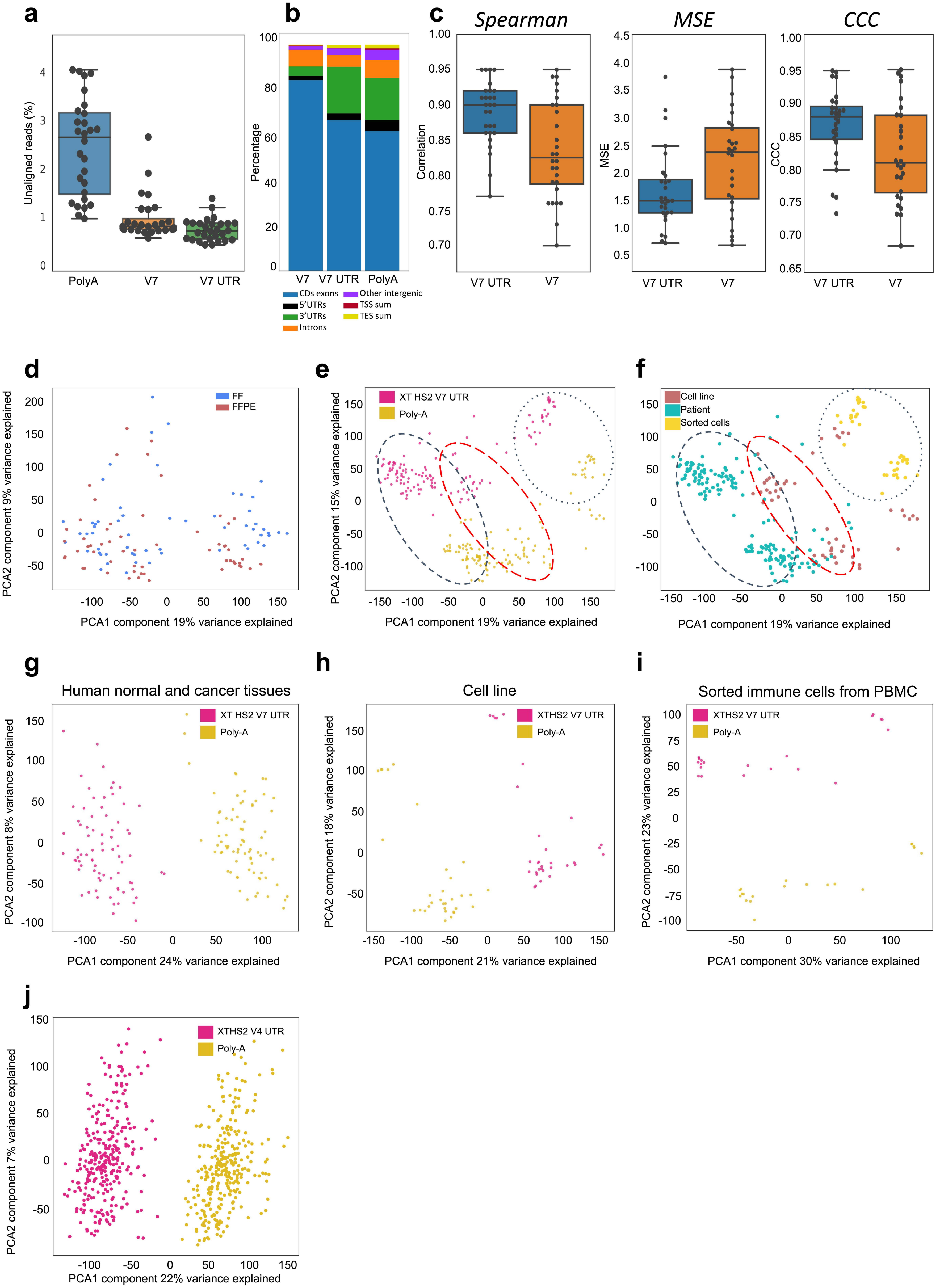
a
Unaligned reads (%)
PolyA
V7
V7 UTR
b
Percentage
V7
V7 UTR
PolyA
CDs exons
5'UTRs
3'UTRs
Introns
Other intergenic
TSS sum
TES sum
c
Spearman
Correlation
MSE
CCC
V7 UTR
V7
d
FF
FFPE
PCA2 component 9% variance explained
PCA1 component 19% variance explained
e
XT HS2 V7 UTR
Poly-A
PCA2 component 15% variance explained
PCA1 component 19% variance explained
f
Cell line
Patient
Sorted cells
PCA1 component 19% variance explained
g
Human normal and cancer tissues
XT HS2 V7 UTR
Poly-A
PCA2 component 8% variance explained
PCA1 component 24% variance explained
h
Cell line
XTHS2 V7 UTR
Poly-A
PCA2 component 18% variance explained
PCA1 component 21% variance explained
i
Sorted immune cells from PBMC
XTHS2 V7 UTR
Poly-A
PCA2 component 23% variance explained
PCA1 component 30% variance explained
j
XTHS2 V4 UTR
Poly-A
PCA2 component 7% variance explained
PCA1 component 22% variance explained

Supplementary Figure 1. Demonstration of batch effects incurred by different sample preparation protocols.

a) Bar plot showing alignment rate of reads to the transcriptome (%) for modified EC and poly-A RNA-seq protocols (N=28 biologically independent samples). For the nested box plots, whiskers indicate 25th percentile (bottom) and 75th percentile (top) +/- 1.5 IQR. **b)** Bar plot showing median fraction of reads (%) aligned to different gene regions grouped by library preparation protocol (N=28 biologically independent samples). **c)** Median Spearman correlation, median Mean Squared Error (MSE) and CCC for V7 and V7 UTR protocols (N=28 biologically independent samples). For the box plots, whiskers indicate 25th percentile (bottom) and 75th percentile (top) +/- 1.5 IQR. **d)** PCA showing the absence of batch effect in FF and FFPE samples sequenced by EC protocol. **e)** PCA on training data showing the batch effect on paired samples sequenced using poly-A RNA-seq vs EC, colored by the protocol, and by (**f**) colored by sample type. **g)** PCA showing the batch effect in EC and poly-A protocols on human tissues, (**h**) cell lines, and (**i**) sorted immune cells from PBMC. **j)** PCA showing the batch effect in MET500 dataset generated using EC Agilent V4 and poly-A protocols. CCC: concordance correlation coefficient

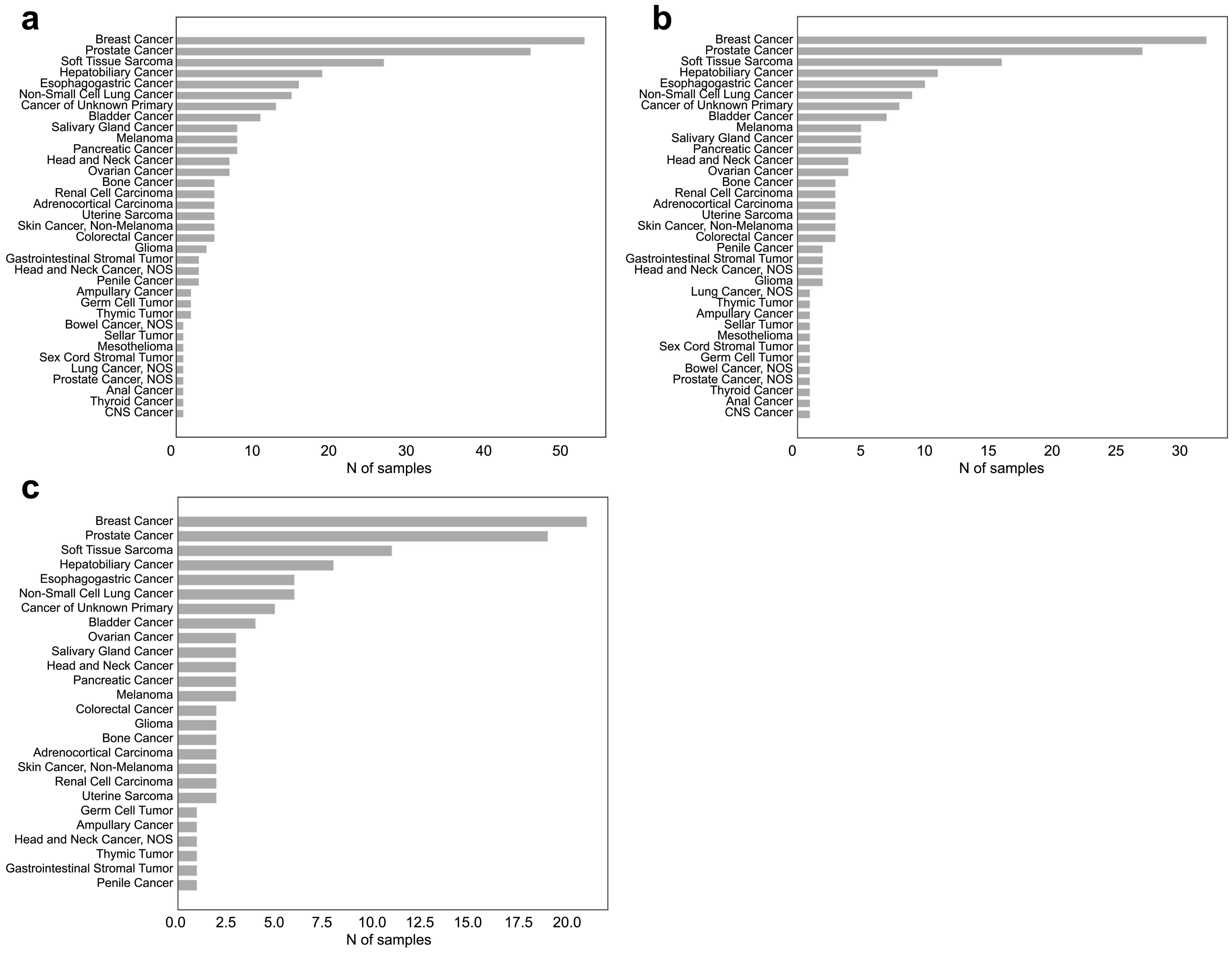

Supplementary Figure 2. The use of the MET500 dataset for training and testing Procrustes
a) Distribution of cancer types in the MET500 dataset. **b)** Training set composition and (**c**) Test (holdout) set composition by cancer types using the MET500 dataset. Panels e and f have been removed because they are no longer relevant to the revised manuscript. Panel d was moved to supplementary figure 9.

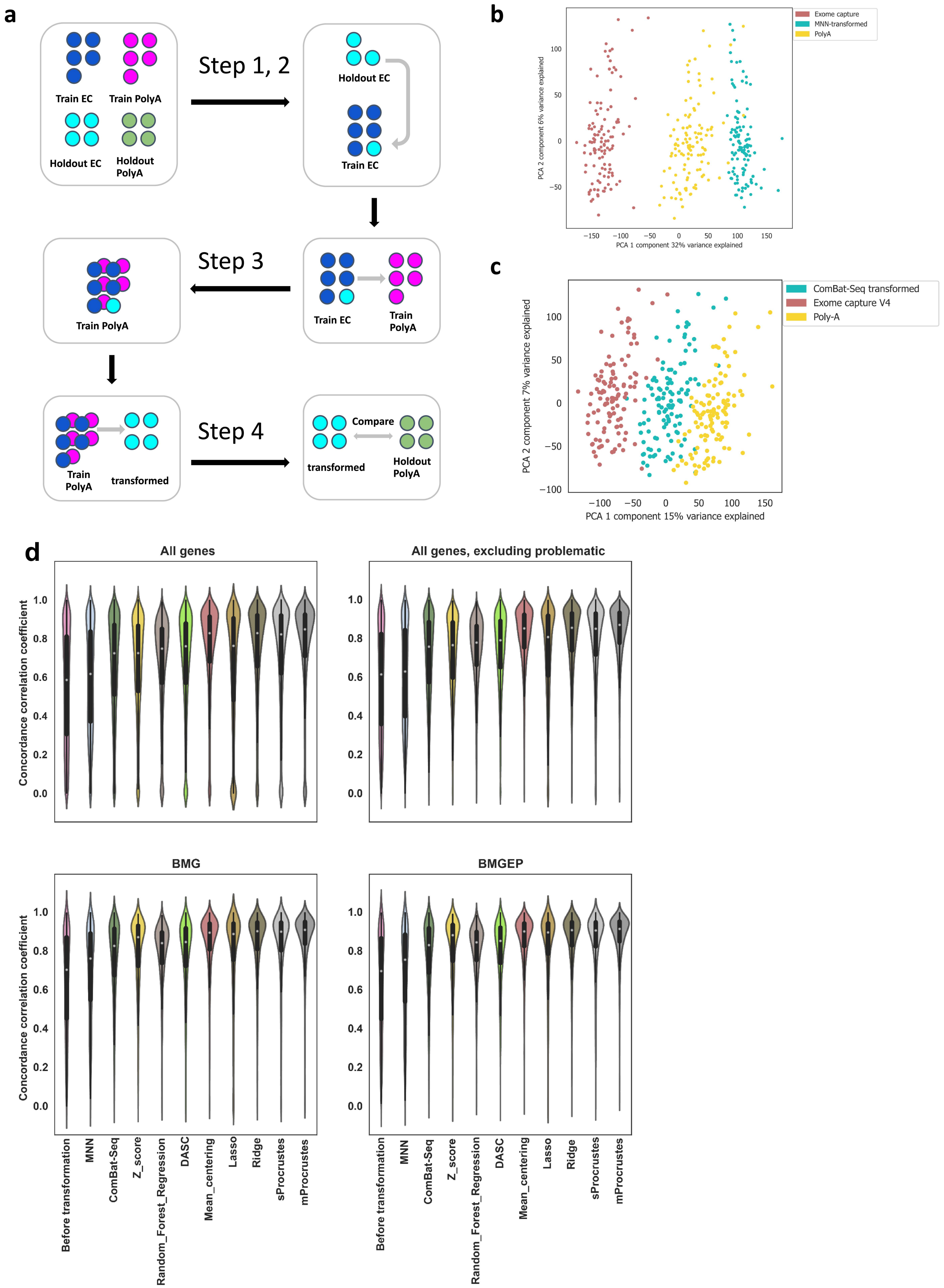
a
Train EC
Train PolyA
Holdout EC
Holdout PolyA
Step 1, 2
Holdout EC
Train EC
Step 3
Train PolyA
Train EC
Train PolyA
Train PolyA
transformed
Step 4
Compare
transformed
Holdout PolyA
b
Exome capture
MNN-transformed
PolyA
PCA 1 component 32% variance explained
PCA 2 component 6% variance explained
c
ComBat-Seq transformed
Exome capture V4
Poly-A
PCA 1 component 15% variance explained
PCA 2 component 7% variance explained
d
All genes
All genes, excluding problematic
BMG
BMGEP
Concordance correlation coefficient
Before transformation
MNN
ComBat-Seq
Z_score
Random_Forest_Regression
DASC
Mean_centering
Lasso
Ridge
sProcrustes
mProcrustes

Supplementary Figure 3. Batch correction by MNN and ComBat-Seq (replaced with ComBat-Seq plot)
a) Schematics showing adjunction holdout-samples to train set for following MNN-transformation. **b)** PCA on MET500-holdout data showing the batch effect after correction of EC-based expression by MNN (N=115 biologically independent samples). **c)** PCA on MET500-holdout data showing the batch effect after correction of EC-based expression by ComBat-Seq (N=115).
d) Violin plots showing benchmarking results measured in CCC values for the barplots in Fig. 3b (N=115 biologically independent samples). BMG: biologically meaningful genes. BMGEP: biologically meaningful genes, excluding problematic genes. For the nested box plots, whiskers indicate 25th percentile (bottom) and 75th percentile (top) +/- 1.5 IQR.

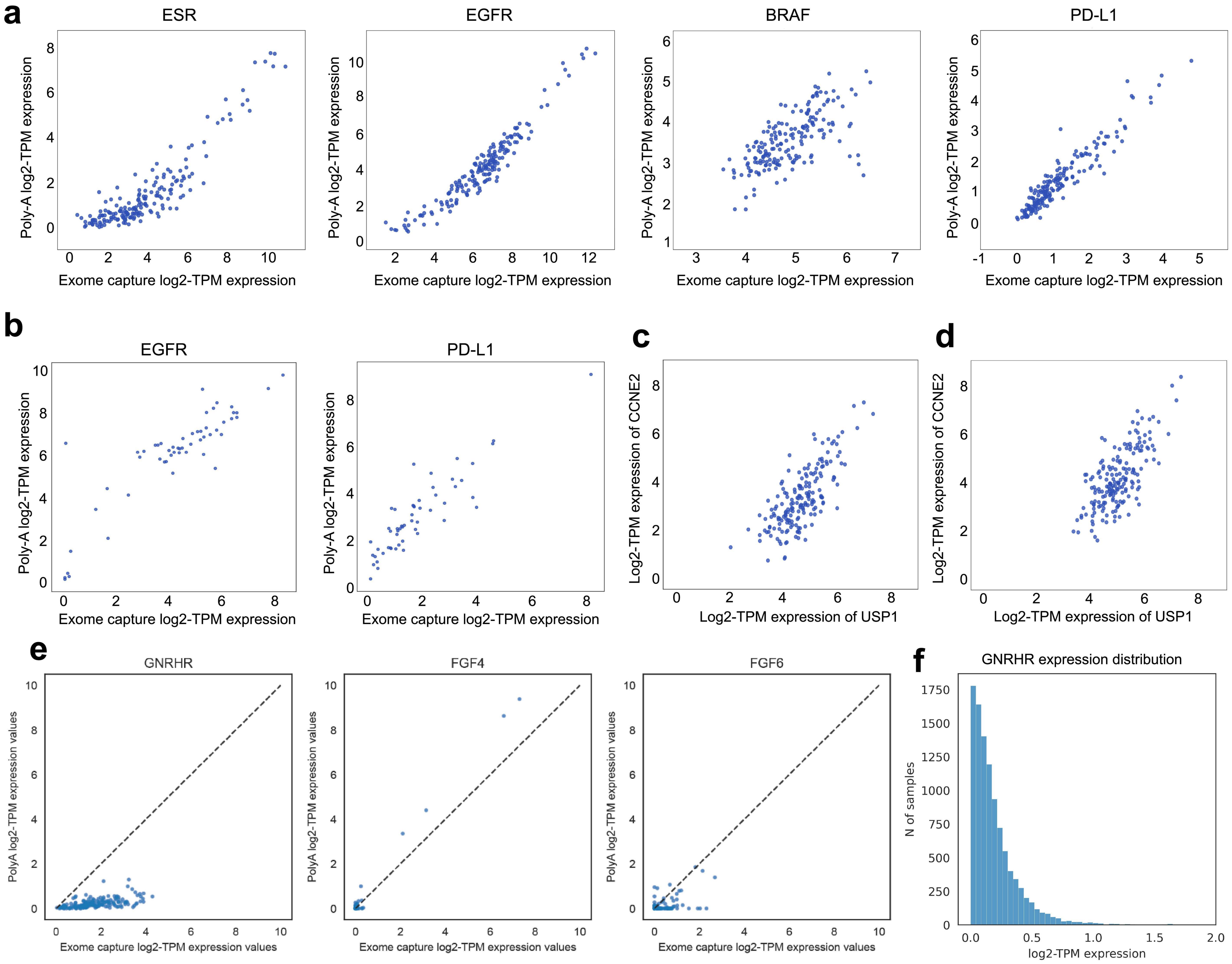

Supplementary Figure 4. Determination of linear correlation between the expression of individual genes and co-expression of specific genes using the MET500 dataset and TCGA cohort (added GNRHR, FGF4, and FGF6 plots)

a) Linear correlation of gene expression between V4 EC and poly-A protocols in MET500 training set. **b)** Linear correlation of gene expression between V7 EC and poly-A protocols (lab data). **c)** Linear correlation of USP1 and CCNE2 in MET500 training set. Pearson correlation equals **(c)** 0.72 and **(d)** 0.74 for EC and poly-A. **e)** Scatter plots for Agilent V4 vs. poly-A RNA-seq data depicting the expression of GNRHR, FGF4, and FGF6 in the MET500 dataset. **f)** Distribution for GNRHR expression in the TCGA cohort (N=10,269 biologically independent samples).

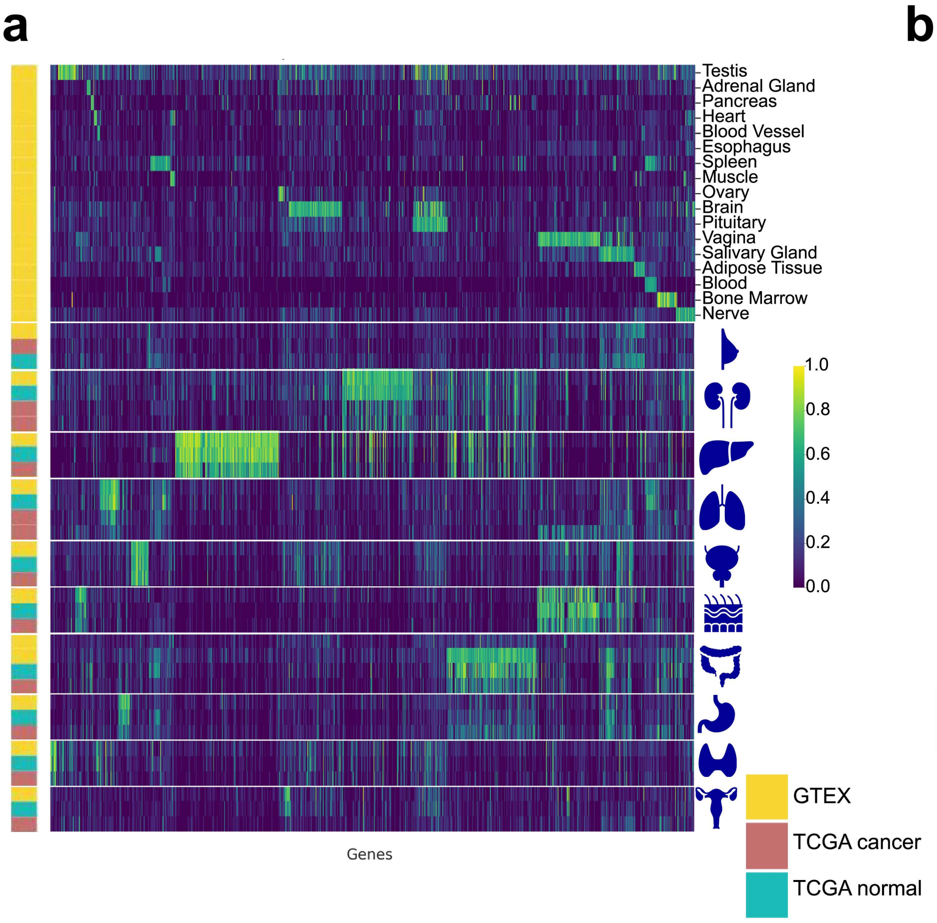

Supplementary Figure 5. Gene group selection

a) Clustered heatmap of tissue specific gene expression. **b)** Workflow for gene group selection.

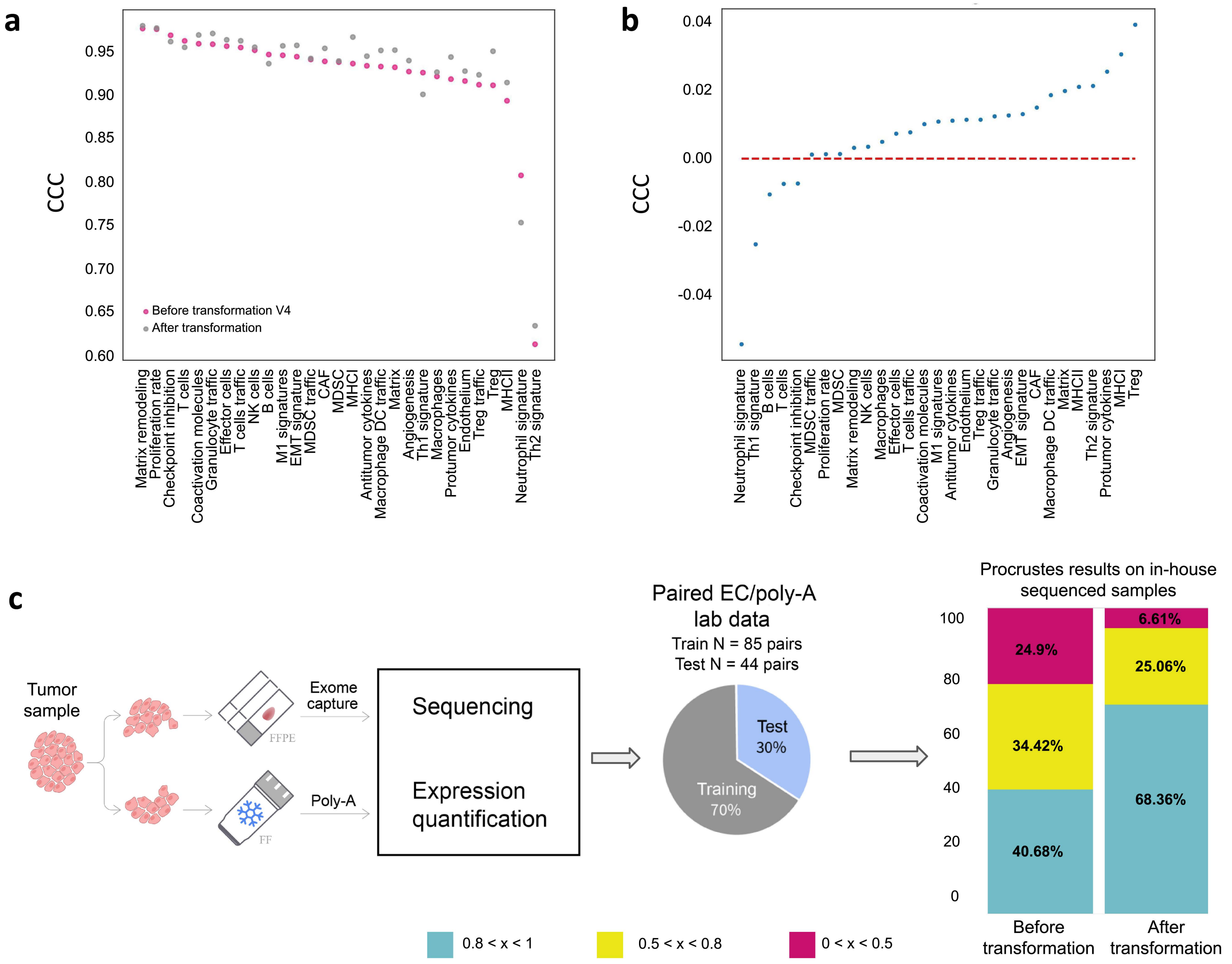

Supplementary Figure 6. Application of Procrustes to tumor microenvironment gene signature.

a) CCC values for microenvironment gene signature (using ssGSEA) and (**b**) CCC value differences for paired Poly-A and EC samples before and after transformation by mProcrustes.

CCC - concordance correlation coefficient

c) Schematics showing our workflow for modeling and validating Procrustes. Each sample was processed in-house, once with the EC-based protocol and once with poly-A RNA-seq protocol.

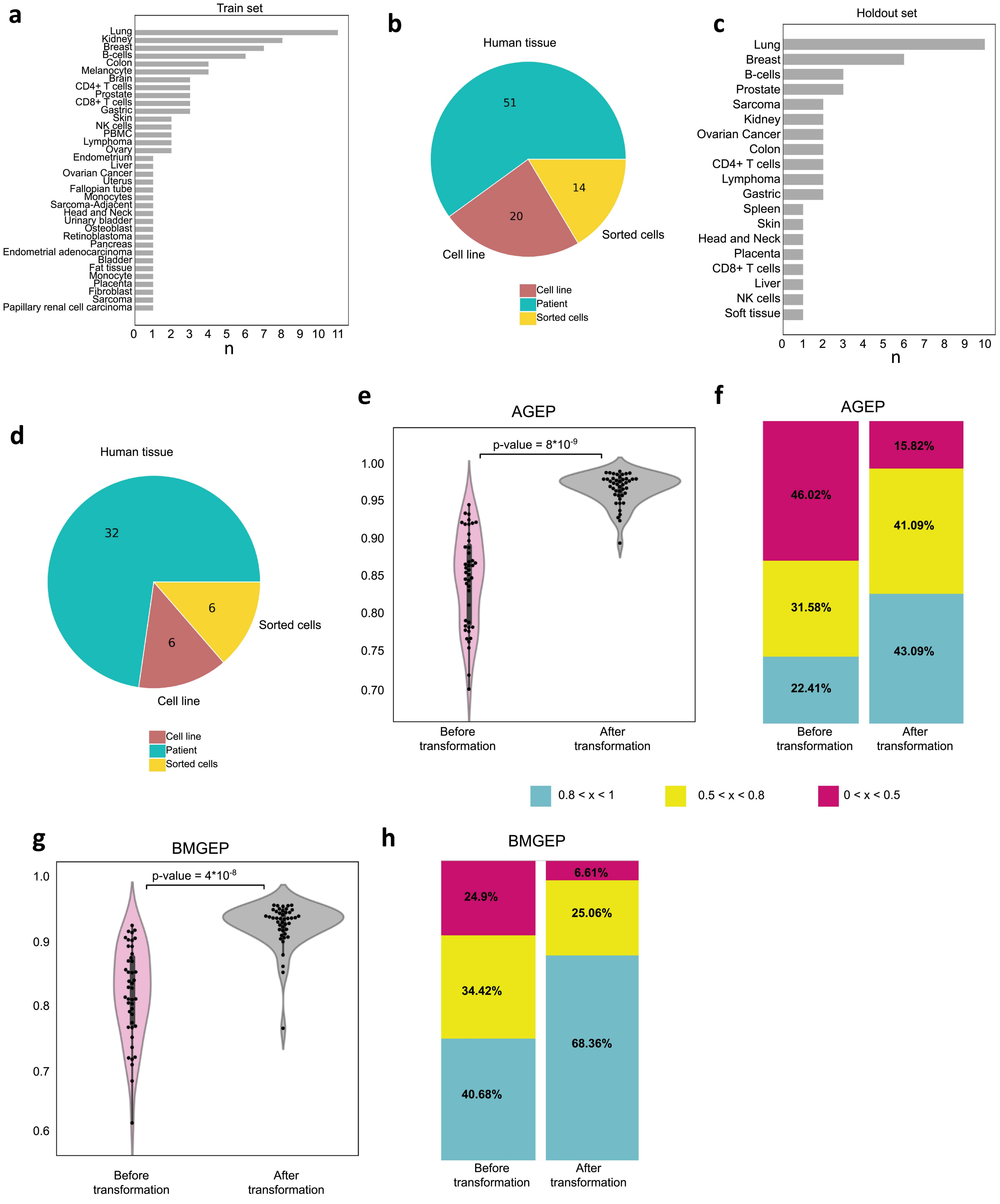
a
Train set
Lung
Kidney
Breast
B-cells
Colon
Melanocyte
Brain
CD4+ T cells
Prostate
CD8+ T cells
Gastric
Skin
NK cells
PBMC
Lymphoma
Ovary
Endometrium
Liver
Ovarian Cancer
Uterus
Fallopian tube
Monocytes
Sarcoma-Adjacent
Head and Neck
Urinary bladder
Osteoblast
Retinoblastoma
Pancreas
Endometrial adenocarcinoma
Bladder
Fat tissue
Monocyte
Placenta
Fibroblast
Sarcoma
Papillary renal cell carcinoma
n
b
Human tissue
51
20
14
Cell line
Sorted cells
Cell line
Patient
Sorted cells
c
Holdout set
Lung
Breast
B-cells
Prostate
Sarcoma
Kidney
Ovarian Cancer
Colon
CD4+ T cells
Lymphoma
Gastric
Spleen
Skin
Head and Neck
Placenta
CD8+ T cells
Liver
NK cells
Soft tissue
n
d
Human tissue
32
6
6
Sorted cells
Cell line
Cell line
Patient
Sorted cells
e
AGEP
p-value = 8*10-9
Before transformation
After transformation
f
AGEP
46.02%
31.58%
22.41%
15.82%
41.09%
43.09%
Before transformation
After transformation
0.8 < x < 1
0.5 < x < 0.8
0 < x < 0.5
g
BMGEP
p-value = 4*10-8
Before transformation
After transformation
h
BMGEP
24.9%
34.42%
40.68%
6.61%
25.06%
68.36%
Before transformation
After transformation

Supplementary Figure 7. Application of Procrustes to lab and clinical data for various gene groups.

a) Train data split by cancer types for lab data. **b)** Proportion of biological subtypes for train lab data. **c)** Test data split by cancer types for clinical samples. **d)** Proportion of biological subtypes for test lab data.

e) Pairwise CCC values before and after transformation in AGEP group (N=44 biologically independent samples). For the nested box plots, whiskers indicate 25th percentile (bottom) and 75th percentile (top) +/- 1.5 IQR. **f)** Performance of mProcrustes on clinical samples in AGEP. CCC: concordance correlation coefficient. Colors reflecting CCC intervals: red $0<x<0.5$; yellow $0.5<x<0.8$; green $0.8<x<1.0$. **g)** Pairwise CCC values before and after transformation in BMGEP group (N=44 biologically independent samples). For the nested box plots, whiskers indicate 25th percentile (bottom) and 75th percentile (top) +/- 1.5 IQR. **h)** Performance of developed algorithms on laboratory data in BMGEP. Colors reflecting CCC intervals: red $0<x<0.5$; yellow $0.5<x<0.8$; green $0.8<x<1.0$.

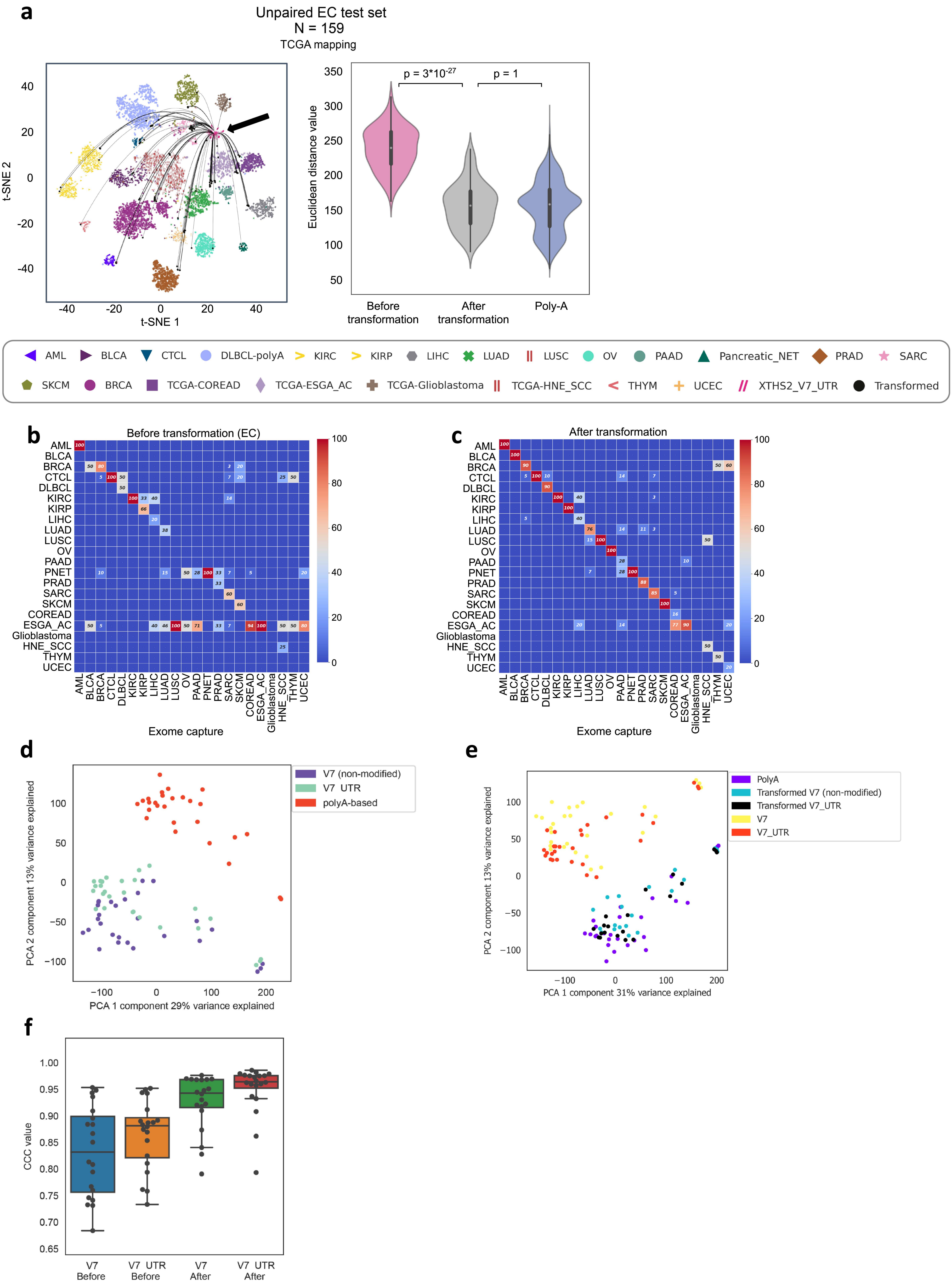
a
Unpaired EC test set
N = 159
TCGA mapping
t-SNE 1
t-SNE 2
Euclidean distance value
p = 3*10-27
p = 1
Before transformation
After transformation
Poly-A
AML
BLCA
CTCL
DLBCL-polyA
KIRC
KIRP
LIHC
LUAD
LUSC
OV
PAAD
Pancreatic_NET
PRAD
SARC
SKCM
BRCA
TCGA-COREAD
TCGA-ESGA_AC
TCGA-Glioblastoma
TCGA-HNE_SCC
THYM
UCEC
XTHS2_V7_UTR
Transformed
b
Before transformation (EC)
c
After transformation
Exome capture
d
V7 (non-modified)
V7 UTR
polyA-based
PCA 1 component 29% variance explained
PCA 2 component 13% variance explained
e
PolyA
Transformed V7 (non-modified)
Transformed V7_UTR
V7
V7_UTR
PCA 1 component 31% variance explained
PCA 2 component 13% variance explained
f
CCC value
V7 Before
V7 UTR Before
V7 After
V7 UTR After

Supplementary Figure 8. Demonstration of concordance between EC-based data and data from the TCGA cohort upon application of Procrustes

a) A total of 159 samples were processed in-house with EC-based protocol only. The resulting data were transformed using Procrustes and then compared to poly-A RNA-seq data for corresponding TCGA cohorts. The black arrow points to the cluster of EC-based test dataset. The black lines radiating from this cluster represent the projection of specific samples onto specific poly-A RNA-seq datasets in corresponding TCGA cohorts. Boxplot represents Euclidean distances from samples to corresponding TCGA cohort before and after transformation with Procrustes. For the nested box plots, whiskers indicate 25th percentile (bottom) and 75th percentile (top) +/- 1.5 IQR.

b and c) Heatmaps showing concordance of diagnoses of clinical samples with target TCGA cohort before and after transformation by mProcrustes.

PCA plots for data from original EC (Agilent XT HS2 V7), modified EC (Agilent XT HS2 V7 UTR), and poly-A RNA-seq protocols, for data comparison before **(d)** and after **(e)** transformation by Procrustes. For the box plots, the whiskers indicate 25th percentile (bottom) and 75th percentile (top) +/- 1.5 IQR.

f) Boxplot of CCC values within EC-poly-A pairs for both V7 and V7 UTR protocols before and after transformation by Procrustes. The samples used to generate these plots are the same as the ones used to generate data for Supplementary Fig. 1a-c (where N = 28). To prevent data leakage, we excluded 8 samples that had already been used to train Procrustes, resulting in a sample size of N = 20 for these plots. For the nested box plots, whiskers indicate 25th percentile (bottom) and 75th percentile (top) +/- 1.5 IQR.

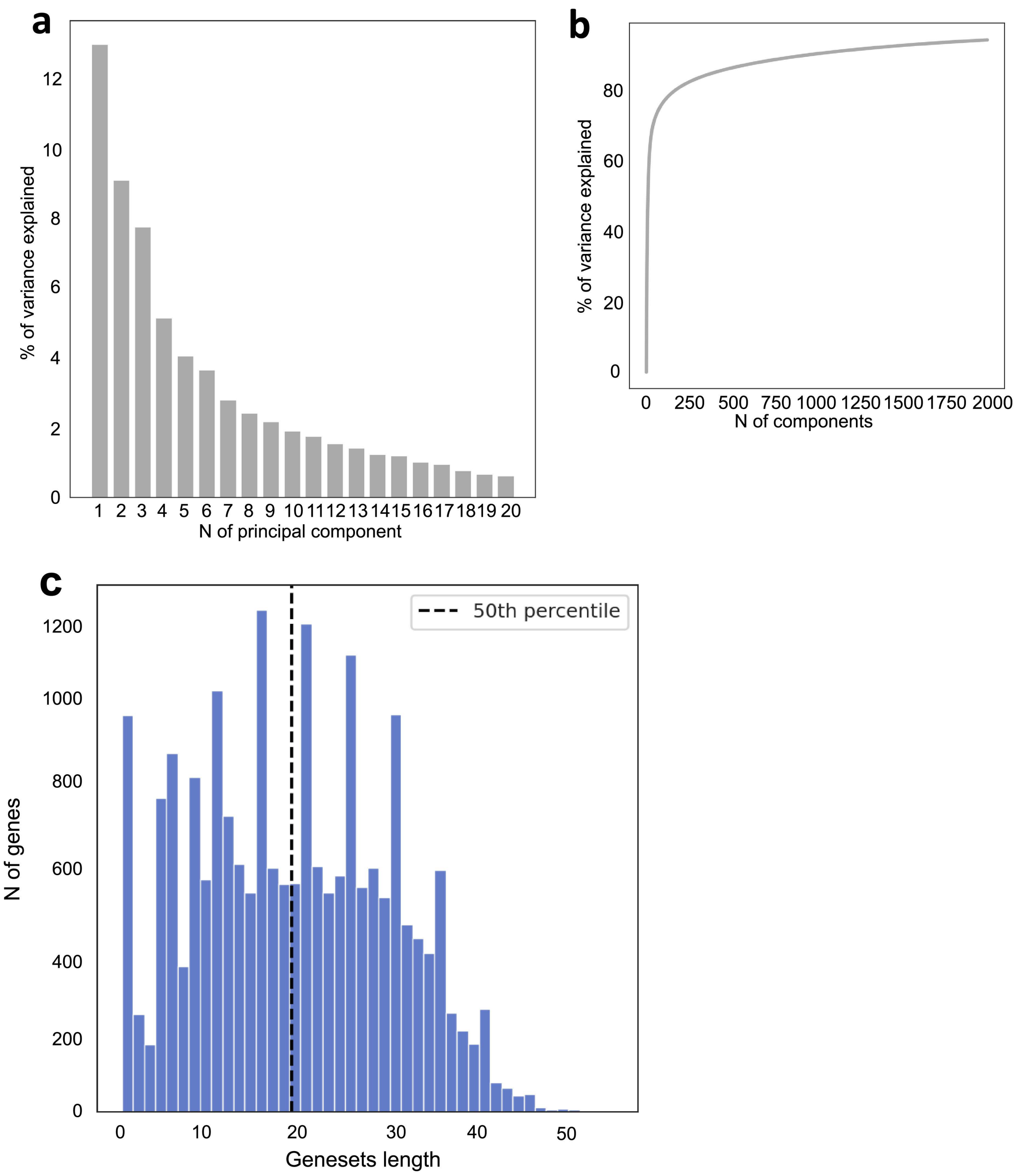

Supplementary Figure 9. Mapping EC-based data to public poly-A cohorts
a) Explained variance for each of the top 20 principal components (PCs) after PCA decomposition of poly-A mapping data. **b)** Cumulative explained variances for poly-A mapping data. **c)** Distribution of geneset lengths for mProcrustes model development.
