# Supplementary material for: Procrustes is a machine-learning approach that removes cross-platform batch effects from clinical RNA sequencing data
Source: Commun Biol. 2024 Mar 30;7:392. doi: 10.1038/s42003-024-06020-z (PMC10981711; doi:10.1038/s42003-024-06020-z)
Supplement: Supplementary file 2 — Description of Supplementary Materials [file 42003_2024_6020_MOESM2_ESM.docx]

**Description of Additional Supplementary Files**

**File name:** Supplementary File 1

**Description:** Exome-capture probes coverage

**File name:** Supplementary Data 1

**Description:** Sample information for comparison of V7 versus V7 UTR EC RNA-seq comparison

**File name:** Supplementary Data 2

**Description:** Samples used for V7, V7_UTR and Poly-A RNA-seq comparison and alignment rates

**File name:** Supplementary Data 3

**Description:** Cell lines used in this study

**File name:** Supplementary Data 4

**Description:** Data annotation for triple FFPE (EC V7 UTR protocol) and FF samples sequenced both using Poly-A and EC V7 UTR protocol

**File name:** Supplementary Data 5

**Description:** FF-FFPE correlation on Exome capture-based RNA-seq

**File name:** Supplementary Data 6

**Description:** MET500 Train and holdout split indexes

**File name:** Supplementary Data 7

**Description:** Gene group definition

**File name:** Supplementary Data 8

**Description:** mProcrustes model gene lists

**File name:** Supplementary Data 9

**Description:** TCGA and GTEx samples used to identify tissue specific genes

**File name:** Supplementary Data 10

**Description:** GTEX and TCGA tissue specific expression gene list

**File name:** Supplementary Data 11

**Description:** MET500 Batch correction comparison results after sProcrustes and mProcrustes transformation

**File name:** Supplementary Data 12

**Description:** MET500 Batch correction comparison results

**File name:** Supplementary Data 13

**Description:** MET500 ssGSEA scores

**File name:** Supplementary Data 14

**Description:** Annotation of clinical samples for assessing Procrustes model clinical utility

**File name:** Supplementary Data 15

**Description:** EC V7 UTR lab data for TCGA mapping using Procrustes

**File name:** Supplementary Data 16

**Description:** Cohorts for mapping
