# Supplementary material for: Procrustes is a machine-learning approach that removes cross-platform batch effects from clinical RNA sequencing data
Source: Commun Biol. 2024 Mar 30;7:392. doi: 10.1038/s42003-024-06020-z (PMC10981711; doi:10.1038/s42003-024-06020-z)
Supplement: Supplementary file 5 — Reporting Summary [file 42003_2024_6020_MOESM5_ESM.pdf]

nature portfolio

Corresponding author(s): Nathan Fowler, M.D.

Last updated by author(s): YYYY-MM-DD

Reporting Summary

Nature Portfolio wishes to improve the reproducibility of the work that we publish. This form provides structure for consistency and transparency in reporting. For further information on Nature Portfolio policies, see our Editorial Policies and the Editorial Policy Checklist.

Statistics

For all statistical analyses, confirm that the following items are present in the figure legend, table legend, main text, or Methods section.

n/a	Confirmed	
☐	☒	The exact sample size (*n*) for each experimental group/condition, given as a discrete number and unit of measurement
☐	☒	A statement on whether measurements were taken from distinct samples or whether the same sample was measured repeatedly
☐	☒	The statistical test(s) used AND whether they are one- or two-sided<br>*Only common tests should be described solely by name; describe more complex techniques in the Methods section.*
☐	☒	A description of all covariates tested
☐	☒	A description of any assumptions or corrections, such as tests of normality and adjustment for multiple comparisons
☐	☒	A full description of the statistical parameters including central tendency (e.g. means) or other basic estimates (e.g. regression coefficient) AND variation (e.g. standard deviation) or associated estimates of uncertainty (e.g. confidence intervals)
☐	☒	For null hypothesis testing, the test statistic (e.g. *F*, *t*, *r*) with confidence intervals, effect sizes, degrees of freedom and *P* value noted<br>*Give P values as exact values whenever suitable.*
☐	☒	For Bayesian analysis, information on the choice of priors and Markov chain Monte Carlo settings
☐	☒	For hierarchical and complex designs, identification of the appropriate level for tests and full reporting of outcomes
☐	☒	Estimates of effect sizes (e.g. Cohen's *d*, Pearson's *r*), indicating how they were calculated

Our web collection on statistics for biologists contains articles on many of the points above.

Software and code

Policy information about availability of computer code

Data collection	No specific software was used to collect the data.
Data analysis	FastQC v0.11.5 (https://www.bioinformatics.babraham.ac.uk/projects/fastqc/), FastQ Screen v0.11.119, RSeQC v3.0.019,20, and MultiQC v1.621 were used to perform quality control (QC) of all NGS samples. Sample correspondence was confirmed by HLA comparison using OptiType22 for RNA-Seq. RNA-Seq reads were aligned to GRCh38.d1.vd1 using Kallisto v0.42.4 and normalized into transcripts per million (TPM). Data visualization was performed using matplotlib (v1.5.1) and seaborn (v0.7.1) for Python. UMAP projection was performed (https://github.com/lmcinnes/umap) and visualized with matplotlib. tSNE projection was performed and visualized with matplotlib (https://github.com/DmitryUlyanov/Multicore-TSNE). Wilcoxon signed-rank test was used to assess the difference between samples (pairwise) before and after transformation by the Procrustes model. Pearson and Spearman correlations were calculated to define co-expressed genes. All statistical tests were performed using the SciPy Python library. Arithmetic mean, median, and standard deviation (STD) were calculated using NumPy. Root mean squared error (RMSE) was calculated for data before and after transformation (within gene) using the scikit- learn Python library (https://scikit-learn.org). Concordance correlation coefficients (CCCs) were calculated according to Lawrence I-Kuei Lin. Principal component analysis (PCA) was performed using randomized singular value decomposition (SVD). For decomposition, the mutual nearest neighbors (MNN) method (https://github.com/chriscainx/mnnpy) was used (Haghverdi et al, 2018). ComBat-Seq (https://bioconductor.org/packages/release/bioc/html/sva.html), DASC (https://github.com/zhanglabNKU/DASC), Ridge and Lasso regression, and Random Forest regression from scikit-learn Python library were chosen as benchmarking targets. NumPy Python library was used to perform Z-score and BMC normalization in benchmarking. ElasticNetCV from scikit-learn Python library was used to make Procrustes.

For manuscripts utilizing custom algorithms or software that are central to the research but not yet described in published literature, software must be made available to editors and reviewers. We strongly encourage code deposition in a community repository (e.g. GitHub). See the Nature Portfolio guidelines for submitting code & software for further information.

Data

Policy information about availability of data

All manuscripts must include a data availability statement. This statement should provide the following information, where applicable:

- Accession codes, unique identifiers, or web links for publicly available datasets
- A description of any restrictions on data availability
- For clinical datasets or third party data, please ensure that the statement adheres to our policy

Sequencing data for this study can be accessed from the NCBI Short Read Archive (SRA) using accession number # (the accession number will be updated once finalized). Accessions for the datasets used in this study are as follows: phs000178 (TCGA), phs000673.v2.p1 (MET500), phs001657.v1.p1 (AML), and GSE98894 (PNET). Some data from this study are not publicly available because disclosing these data would compromise the privacy of research participants and violate their consent.

Research involving human participants, their data, or biological material

Policy information about studies with human participants or human data. See also policy information about sex, gender (identity/presentation), and sexual orientation and race, ethnicity and racism.

Reporting on sex and gender	This informations has not been collected.
Reporting on race, ethnicity, or other socially relevant groupings	This information has not been collected.
Population characteristics	This information has not been collected.
Recruitment	Not applicable
Ethics oversight	Clinical samples were included in this study. Each patient provided informed consent. The use of clinical samples was conducted in accordance with the Declaration of Helsinki and has been granted exemption from ethics approval by the Biomedical Research Alliance of New York (BRANY) Institutional Review Board (IRB) (BRANY study #22-12-938-853).

Note that full information on the approval of the study protocol must also be provided in the manuscript.

Field-specific reporting

Please select the one below that is the best fit for your research. If you are not sure, read the appropriate sections before making your selection.

☒ Life sciences ☐ Behavioural & social sciences ☐ Ecological, evolutionary & environmental sciences

For a reference copy of the document with all sections, see nature.com/documents/nr-reporting-summary-flat.pdf

Life sciences study design

All studies must disclose on these points even when the disclosure is negative.

Sample size	No prior sample-size calculation was performed. However, to make a model with in-house sequenced samples, we targeted to acquire as many samples as possible, considering the need for diverse cancer types to achieve a wide range of gene expression distribution. Also, we performed a post hoc statistical power analysis using "FTestAnovaPower" function from the statsmodels Python library. We considered using a "Standardized mean difference" approach for effect size calculation, which resulted in 0.56 effect size value for concordance correlation coefficient before and after application of Procrustes. For alpha=0.0001 and power=0.95, we obtained a value of 108 for optimal sample size. Our final sample size of 129 samples processed in-house exceeds this value.
Data exclusions	Samples were excluded from RNA-seq analysis based on quality control (QC) criteria (e.g., having a low number of reads that aligned or that failed QC analysis by FastQC and FastQ Screen).
Replication	Replications for this study were not performed due to a limited amount of tissue from acquired samples.
Randomization	Only allocation of samples was performed when splitting data into training and holdout (test) sets. Splitting was performed using train_and_test_split function from the scikit-learn Python library with stratification by cancer/tissue types.
Blinding	Blinding was not possible during allocation of samples because randomization was performed based on stratification by cancer/tissue type.

Reporting for specific materials, systems and methods

We require information from authors about some types of materials, experimental systems and methods used in many studies. Here, indicate whether each material, system or method listed is relevant to your study. If you are not sure if a list item applies to your research, read the appropriate section before selecting a response.

Materials & experimental systems

n/a	Involved in the study
☒	☐ Antibodies
☐	☒ Eukaryotic cell lines
☒	☐ Palaeontology and archaeology
☒	☐ Animals and other organisms
☒	☐ Clinical data
☒	☐ Dual use research of concern
☒	☐ Plants

Methods

n/a	Involved in the study
☒	☐ ChIP-seq
☒	☐ Flow cytometry
☒	☐ MRI-based neuroimaging

Eukaryotic cell lines

Policy information about cell lines and Sex and Gender in Research

Cell line source(s)	Cell lines used are listed in Supplementary Data 13. All cell lines were purchased from ATCC except GM12877 and GM12878, which were purchased from Coriell Institute for Medical Research.
Authentication	None of the cell lines used were authenticated in our laboratory.
Mycoplasma contamination	According to ATCC, mycoplasma is not detected in the cell lines we procured. For cell lines procured from Coriell Institute for Medical Research, information concerning mycoplasma contamination is not available, and no further testing was performed in our laboratory.
Commonly misidentified lines (See ICLAC register)	BT-20, RT4, and U-118. All cell lines were used to compare gene expression levels as obtained by different RNA-seq protocols (technical comparison). No biological conclusions were derived from these findings.

Plants

Seed stocks	*Report on the source of all seed stocks or other plant material used. If applicable, state the seed stock centre and catalogue number. If plant specimens were collected from the field, describe the collection location, date and sampling procedures.*
Novel plant genotypes	*Describe the methods by which all novel plant genotypes were produced. This includes those generated by transgenic approaches, gene editing, chemical/radiation-based mutagenesis and hybridization. For transgenic lines, describe the transformation method, the number of independent lines analyzed and the generation upon which experiments were performed. For gene-edited lines, describe the editor used, the endogenous sequence targeted for editing, the targeting guide RNA sequence (if applicable) and how the editor was applied.*
Authentication	*Describe any authentication procedures for each seed stock used or novel genotype generated. Describe any experiments used to assess the effect of a mutation and, where applicable, how potential secondary effects (e.g. second site T-DNA insertions, mosiacism, off-target gene editing) were examined.*
